# Using a practical molecular capsular serotype prediction strategy to investigate *Streptococcus pneumoniae* serotype distribution and antimicrobial resistance in Chinese local hospitalized children

**DOI:** 10.1186/s12887-016-0589-7

**Published:** 2016-04-26

**Authors:** Ping Jin, Lijuan Wu, Shahin Oftadeh, Timothy Kudinha, Fanrong Kong, Qiyi Zeng

**Affiliations:** Pediatric Center, Zhujiang Hospital, Southern Medical University, Guangzhou, 510282 P. R. China; Paediatric Intensive Care Unit, Bao’an Maternity & Child Health Hospital affiliated with Jinan University, Shenzhen, P. R. China; Department of Clinical Laboratory, Bao’an Maternity & Child Health Hospital affiliated with Jinan University, Shenzhen, P. R. China; Centre for Infectious Diseases and Microbiology Laboratory Services, ICPMR – Pathology West, University of Sydney, Westmead Hospital, Darcy Road, Westmead, NSW Australia; Charles Sturt University, Leeds Parade, Orange, NSW Australia

**Keywords:** *Streptococcus pneumoniae*, serotype prediction, *cpsB* sequetyping, Sequential multiplex PCR, Antibiotic multidrug resistance

## Abstract

**Background:**

China is one of ten countries with the highest prevalence rate of pneumococcal infections. However, there is limited serotype surveillance data for *Streptococcus pneumoniae*, especially from the community or rural regions, partly due to limited serotyping capacity because Quellung serotyping is only available in few centers in China. The aim of this study was to develop a simple, practical and economic pneumococcal serotype prediction strategy suitable for future serotype surveillance in China.

**Methods:**

In this study, 193 *S. pneumoniae* isolates were collected from hospitalized children, 96.9 % of whom were < 5 years old. The *cpsB* sequetyping, complemented by selective and modified USA CDC sequential multiplex-PCR, was performed on all the isolates, and serotypes 6A-6D specific PCRs were done on all serogroup 6 isolates. Based on systematic analysis of available GenBank *cpsB* sequences, we established a more comprehensive *cpsB* sequence database than originally published for *cpsB* sequetyping. Antibiotic susceptibility of all isolates was determined using the disk diffusion or E-test assays.

**Results:**

We built up a comprehensive *S. pneumoniae* serotype *cpsB* sequetyping database for all the 95 described serotypes first, and then developed a simple strategy for serotype prediction based on the improved *cpsB* sequetyping and selective multiplex-PCR. Using the developed serotype prediction strategy, 191 of 193 isolates were successfully “serotyped”, and only two isolates were “non-serotypeable”. Sixteen serotypes were identified among the 191 “serotypeable” isolates. The serotype distribution of the isolates from high to low was: 19 F (34.7 %), 23 F (17.1 %), 19A (11.9 %), 14 (7.3 %), 15B/15C (6.7 %), 6B (6.7 %), 6A (6.2 %), 9 V/9A (1.6 %); serotypes 6C, 3, 15 F/15A, 23A and 20 (each 1.1 %); serotypes 10B, 28 F/28A and 34 (each 0.5 %). The prevalence of parenteral penicillin resistance was 1.0 % in the non-meningitis isolates and 88.6 % in meningitis isolates. The total rate of multidrug resistance was 86.8 %.

**Conclusions:**

The integrated *cpsB* sequetyping supplemented with selective mPCR and serotypes 6A-6D specific PCRs “cocktail” strategy is practical, simple and cost-effective for use in pneumococcal infection serotype surveillance in China. For hospitalized children with non-meningitis penicillin-susceptible pneumococcal infections, clinicians still can use narrow-spectrum and cheaper penicillin, using the parenteral route, rather than using broader-spectrum and more expensive antimicrobials.

**Electronic supplementary material:**

The online version of this article (doi:10.1186/s12887-016-0589-7) contains supplementary material, which is available to authorized users.

## Background

*Streptococcus pneumoniae* is a leading cause of bacterial pneumonia, meningitis, and sepsis in children worldwide. Although China is among the ten countries with the highest prevalence of pneumococcal cases [1], there is limited epidemiological data on invasive pneumococcal disease in mainland China. Vaccination, targeting the pneumococcal polysaccharide capsule, is the best way to prevent pneumococcal disease, especially in children. The 7-valent pneumococcal conjugate vaccine (PCV7), which is no longer available, became accessible for the private sector in China in September 2008 [2], but was never part of the universal immunization program in this country. Even in Shenzhen (one of the biggest cities in China which borders Hong Kong), the PCV7 immunization rate is still less than 1 % [3].

The capsular polysaccharide is the main virulence determinant of *S. pneumoniae*, and structural differences of this polysaccharide, can divide *S. pneumoniae* into many serotypes. After including the newly identified serotypes 6D, 6E and 11E, there are 46 different serogroups and 95 serotypes of *S. pneumoniae* that have been described to date [4–6]. Conventional serotyping by the Quellung reaction is complex, costly, and requires highly skilled personnel. On the other hand, latex agglutination is a simple and efficient alternative method to Quellung reaction serotyping, but still needs further work to improve its capacity to detect colonizing pneumococcal strains at low density [7]. In recent years, a variety of DNA-based methods that rely on the capsular polysaccharide synthesis locus for the detection of pneumococcal serotypes, have been described, including approaches based on sequencing, restriction fragment length polymorphisms, hybridization assays, microarrays, and different PCR strategies [8–15]. For many developing countries including China, it is crucial to find a practical, simple and cost-effective strategy for routine serotype prediction and pneumococcal serogroup/serotype surveillance.

In a previous study, Leung and collaborators used a single PCR sequencing method targeting *cpsB* gene (sequetyping) to identify *S. pneumoniae* serotypes [13]. The USA Centers for Disease Control and Prevention (CDC) has published a sequential multiplex PCR (mPCR) protocol, which, although is the most commonly used molecular assay for identification of *S. pneumoniae* serotypes, is complicated by the need to perform eight sets of multiplex PCRs. Here, we employed *cpsB* sequetyping coupled with local based selective and modified sequential multiplex PCR, and serotypes 6A-6D specific PCRs, to predict the serotypes of 193 *S. pneumoniae* isolates from hospitalized children with pneumococcal infection in our district hospital.

The aim of this study was, as a showcase, to investigate the best combination of the aforementioned methods for use as an initial serotype screening method especially for developing countries. Furthermore, in order to provide some local epidemiological data for current and future planning purposes, we studied the serotype distribution, antibiotic susceptibility and clinical presentation, amongst the 193 *S. pneumoniae* isolates.

## Methods

### *S. pneumoniae* isolates

*S. pneumoniae* isolates (*n* =193) from children, were provided by Shenzhen Bao’an Maternity & Child Health Hospital, during the period January 2009 to December 2013. The identity of the isolates was confirmed using standard microbiological tests, including colony morphology, optochin susceptibility and bile solubility.

Among the 193 isolates, 169 (87.6 %) isolates were from sputum, 17 (8.8 %) from blood, 3 (1.6 %) from pleural fluid, 2 (1.0 %) from cerebrospinal fluid and 2 (1.0 %) from other normally sterile body sites (Additional file 1: Table S1). All the children with pneumonia imply satisfied the World Health Organization standard definition for pneumonia, including classification as non-severe, severe and very severe pneumonia [16]. The serotypes of all the isolates were unknown at the time of receipt and testing. When two isolates from the same subject had an identical serotype, only one isolate was included in the study.

In children with pneumonia (severe or non-severe), sputum was collected with a small suction catheter, which was passed through the nose into the laryngopharynx. The length of the catheter into the respiratory tract was equal to the distance from the apex of the nose to the earlobe, and then to the thyroid cartilage. Upon eliciting a cough reflex, respiratory tract secretions were aspirated. In patients with very severe pneumonia who were under mechanical ventilation, sputum was collected from an endotracheal tube. The squamous epithelial cell numbers of <10 per 10 x objective microscopic field was used as an indicator of good quality sputum for culture [17], and only samples that met this quality criteria were cultured.

The study was approved by the Medical Ethics Committee of Shenzhen Bao’an Maternity & Child Health Hospital affiliated with Jinan University (No. S-2013002); and signed informed consents were obtained from patient’s parents or guardians.

### DNA extraction from bacterial isolates

Pneumococcal isolates were retrieved from storage by subculture on blood agar plates (Columbia II agar base supplemented with 5 % horse blood) and incubated overnight at 37 °C in 5 % CO_2_. Genomic DNA was extracted from bacteria using the AxyGenamp DNA Mini Extraction Kit (Axygen, USA) according to the manufacturer’s instructions, and the purified DNA was diluted in a final volume of 100 μL Tris EDTA buffer and stored at − 20 °C until use.

### Building up a comprehensive 95 serotypes *cpsB* sequetyping database based on GenBank sequences (Additional file 2: Figure S1)

The previous *cpsB* sequetyping database designed by Leung et al. [13] didn’t include all the 95 serotype *cpsB* sequences. Our aim was to extend this work by including all the serotypes described to date in a new sequetyping database. All the *S. pneumoniae* sequences in the GenBank that contained the full-length of *cpsB* (as of Jan 1, 2015) were downloaded, and after sequence alignment using ClustalW and/or Blastn, each GenBank sequence was given a unique *cpsB* sequetype name (see Additional file 3: Table S2 and Additional file 2: Figure S1), ensuring that all GenBank sequences with the same *cpsB* sequence, were given the same sequetype name. The given *cpsB* sequetype name was chosen to reflect the specific serotype (if only one serotype had the sequence) or combination of serotypes (when more than one serotype have a common sequence) it represented. If multiple GenBank sequences had an identical *cpsB* sequetype, only one GenBank sequence was selected as reference to represent the sequetype (Additional file 3: Table S2). All sequences for the 90 Statens Serum Institut serotype reference strains were used as references for the relevant serotypes/sequetypes, whilst for the other sequetype references, we used those from Leung et al. [13] or other publications, and those with longer *cps* gene cluster sequences, because they were well-characterized compared with the other GenBank sequences (Additional file 2: Figure S1). As shown in Additional file 3: Table S2, if any of the reference sequences were longer than *cpsB* sequences (732-bp), the position of full length of *cpsB* (732-bp) was clearly shown on the GenBank sequences. Based on our database, all of the *S. pneumoniae* full-length *cpsB* GenBank sequences (as of Jan 1, 2015) with known serotypes and sequetypes were included (see Additional file 3: Table S2 & Additional file 2: Figure S1).

### The *cpsB* sequetyping workflow for our local isolates (Fig. 1)

**Fig. 1:**
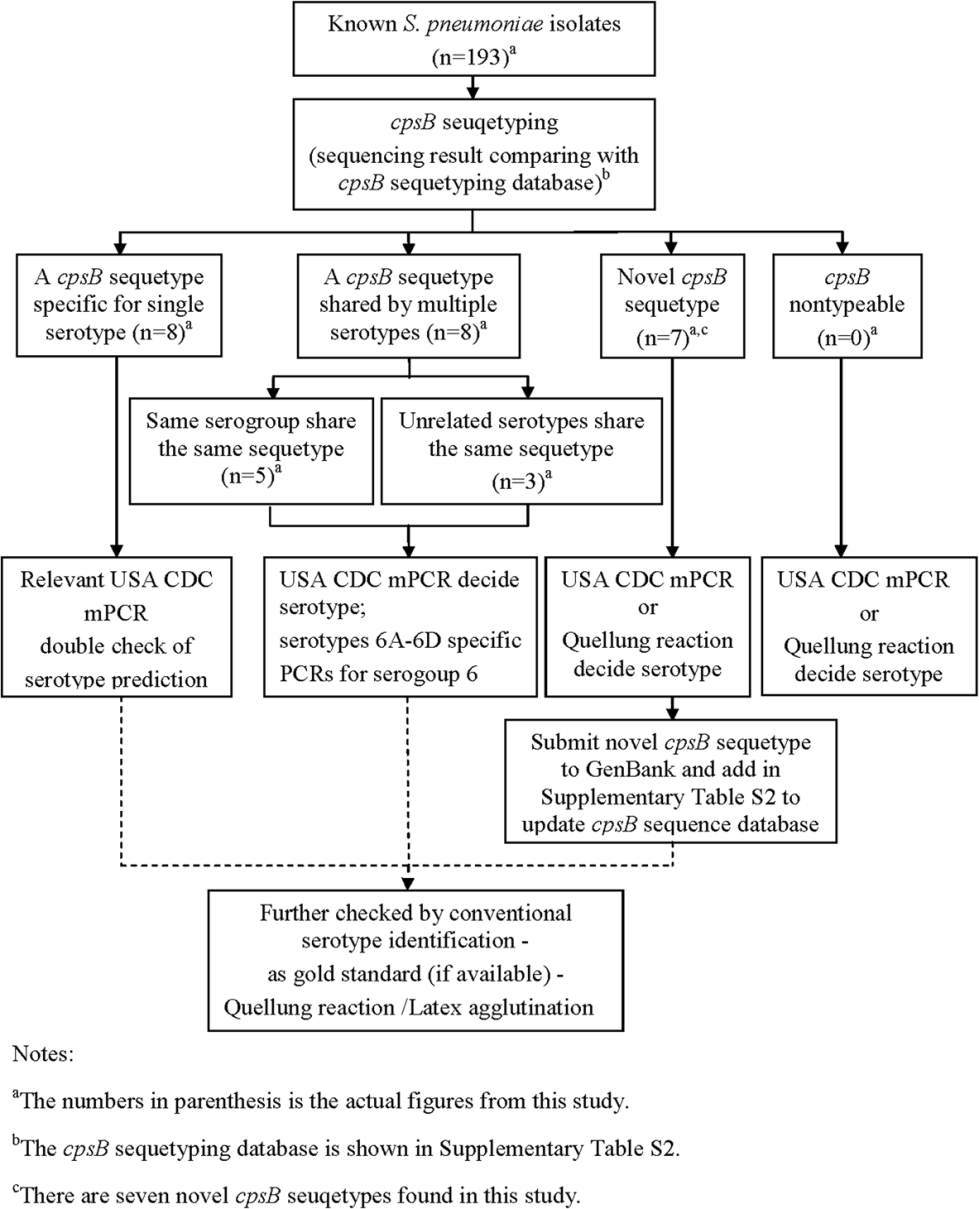
The *Streptococcus pneumoniae* serotype prediction algorithm – a strategy based on *cpsB* sequetyping and selected mPCR. Step 1. The *cpsB* sequetyping was performed on all 193 *S. pneumoniae* isolates. Sequencing results compared with *cpsB* sequetyping database. 21 different sequetypes were identified; included serotype-specific sequetypes, sequetypes shared by multiple serotypes and novel *cpsB* sequetypes. Step 2. Modified and selected USA CDC sequential multiplex PCR to double check, or resolve discrepant results, or identify those that shared the same *cpsB* sequetype. Step 3. Serotypes 6A-6D specific PCRs were performed for serogroup 6 isolates. Step 4. Submit all novel *cpsB* sequetypes to GenBank and update *cpsB* sequetyping database

The *cpsB* sequetyping was performed on all 193 *S. pneumoniae* isolates as previously described [13] using our newly designed comprehensive *cpsB* sequetyping database, as shown in the workflow algorithm in Fig. 1. In brief, as described by Leung et al. [13], a region spanning the *cpsB* gene was amplified by single PCR, the amplicon purified, and the nucleotide sequence determined by double strand sequencing. The amplicon nucleotide sequences were then used to Blastn GenBank database, and if it was identical to any one or more of the GenBank sequences, the serotype (s)/*cpsB* sequetype was decided according to our *cpsB* sequetyping database (see Additional file 3: Table S2). Any mismatch between the sequences and reference sequences in GenBank were manually checked to ascertain the mismatch. Furthermore, USA CDC sequential multiplex PCRs were performed on all 193 *S. pneumoniae* isolates, and serotypes 6A-6D specific PCRs were performed on all serogroup 6 isolates. The results were checked against *cpsB* sequetyping results, before submitting the new sequences to GenBank (Additional file 4: Table S3).

### Sequential multiplex PCR and local data based primer sets selection

Sequential multiplex PCR, as per CDC scheme, was employed to predict the isolate serotype(s) by targeting serotype-specific *cps* regions [11]. The primer sequences, PCR reactions and product detection are as published by the CDC and were updated in Feb 2014. (http://www.cdc.gov/streplab/pcr.html). We further designed combinations of primer sets in the first three reactions to identify the six most predominant serotypes (19 F, 19A, 14, 23 F, 15, 6) in China based on previous studies [18, 19], and three PCV7 vaccine serotypes (4, 9 V/9A, 18). In these modified reactions, reaction 1 contained primers for serotypes 4, 6 and 18; reaction 2 contained primers for serotypes 14, 9 V/9A, 15 F/15A and 19A; and reaction 3 contained primers for serotypes 15B/15C, 19 F and 23 F. If a sample was negative in the first three reactions, eight sequential multiplex PCR reactions were performed as previously described in CDC (USA) webpage (http://www.cdc.gov/streplab/pcr.html).

### Serotypes 6A-6D specific PCRs

In a previous study, we developed serotype-specific PCR to identify serotypes 6A, 6B, 6C and 6D [4]. Using the same protocol and primers, sequential single PCRs were performed with three primer sets to distinguish these serotypes.

### Antimicrobial susceptibility testing

In vitro susceptibility tests were performed using disk diffusion and the following antibiotics were used; erythromycin, clindamycin, levofloxacin, vancomycin, tetracycline, sulfamethoxazole-trimethoprim and chloramphenicol (Oxoid, UK). The minimum inhibitory concentrations for penicillin and ceftriaxone were determined by E-test (AB Biodisk, Solna, Sweden). All tests were performed following the United States Clinical and Laboratory Standards Institute (CLSI) recommendations, and CLSI M100-S25 version of the antibiotic susceptibility breakpoints for *S. pneumoniae* was adopted as criteria for determining drug resistance [20]. *S. pneumoniae* ATCC 49619 was used as the quality-control strain. Isolates not susceptible to three or more classes of antimicrobials were considered multidrug-resistant (MDR)*.*

### Statistical analysis

Data on serotype distribution of the isolates were analyzed using SPSS version 13.0 (SPSS Inc., Chicago, IL) statistical software. Association between serotypes and clinical presentation was tested using χ^2^ test or Fisher’s exact test. A two-tailed cutoff of *P* < 0.05 was considered statistically significant.

## Results

### Clinical data

During the study period, 193 non-duplicate *S. pneumoniae* isolates were collected from hospitalized children with pneumococcal infections. The patients included 121 boys and 72 girls; 126 (65.3 %) children were < 2 years old; 61 (31.6 %) were 2–5 years old; and 6 (3.1 %) were > 5 years old. The distribution of cases by clinical presentation was as follows: non-severe pneumonia (134, 69.4 %), severe and very severe pneumonia (38, 19.7 %), primary bacteremia (17, 8.8 %), meningitis (2, 1 %), urinary tract infections (1, 0.5 %) and cellulitis (1, 0.5 %) (Additional file 1: Table S1).

### Development of reference *cpsB* sequence sequetyping database

We developed a comprehensive *S. pneumoniae* serotype *cpsB* sequence (732-bp) sequetyping reference database for all the 95 described serotypes, including all 390 available GenBank sequences with full length of the *cpsB* sequence name (see Additional file 3: Table S2, Additional file 2: Figure S1). When the same sequetype was shared by two or more serotypes, the sequetype name included all the different serotypes in ascending numerical order (e.g., 24 F-24B-33 F-33A-35A-1).

### The *cpsB* sequetyping results for studied clinical isolates

All the 193 isolates included in the study could be amplified by *cpsB* PCR and yielded satisfactory sequencing results (Table 1, Additional file 4: Table S3). Based on *cpsB* sequence heterogeneity at one or more sites for all isolates, 21 different sequetypes were identified: eight serotype-specific sequetypes could predict isolates to serotype level (3, 9 V, 6B, 10B, 14, 19A, 23 F, 23A); five sequetypes shared by different serotypes but in the same serogroups (6C-6D-1, 6B-6E-6X-1, 15B-15C-1, 19 F-19A-1, and 28 F-28A-1) could predict isolates to the serogroup level; and three sequetypes shared by different serotypes - serotypes 13 and 20, 15A and 33B, 17A and 34, could not be differentiated from each other. Of the 193 isolates tested, 66 (34.2 %) were sequetyped to the serotype level and 107 (55.4 %) to the serogroup level.

**Table 1:** Serotype distribution among 193 *S. pneumoniae* isolates as determined by *cpsB* sequetyping and selected sequential mPCR

Serotype/Serogroup	No. of isolates with serotypes determined by		Novel sequetypes
	mPCR/6A-6D specific PCR	*cpsB* sequetyping^a^	
3	3 (*n* = 2)	1 bp-3-2 (*n* = 2)	3-sz-1 (3–5)
6A	6/6A (*n* = 12)	2 bp-6C-6D-1 (*n* = 12)	6A-sz-1 (6A-5)
6B	6/6B (*n* = 13)	6B-1 (*n* = 1) 6B-6E-6X-1 (*n* = 12)	
6C	6/6C (*n* = 2)	6C-6D-1 (*n* = 2)	
9 V/9A	9 V/9A (*n* = 3)	9 V-1 (*n* = 3)	
10B	unknown (*n* = 1)^d^	10B-1 (*n* = 1)	
14	14 (*n* = 14)	14-1 (*n* = 14)	
15 F/15A	15 F/15A (*n* = 2)	3 bp-15A-33B-1 (*n* = 2)^b^	15 F/15A-sz-1(15 F-15A-1)
15B/15C	15B/15C (*n* = 13)	15B-15C-1 (*n* = 13)	
19 F	19 F (*n* = 67)	19 F-19A-1 (*n* = 67)	
19A	19A (*n* = 23)	19A-2 (*n* = 23)	
20	20 (*n* = 2)	13-20A-20B-1 (*n* = 2)^b^	
23 F	23 F (*n* = 33)	23 F-1 (*n* = 20) 11 bp-11 F-1 (*n* = 9)^c^ 6A-6B-6 F (*n* = 2)^c^ 2 bp-6A-6B-6 F-1 (*n* = 1)^c^ 13-20A-20B (*n* = 1)^c^	23 F-sz-1 (23 F-2) 23 F-6A-6B-6 F-sz-1 23 F-sz-2 (23 F-2) 23 F-13-20A-20B-1
23A	23A (*n* = 2)	23A-1 (*n* = 2)	
28 F/28A	unknown (*n* = 1)^d^	28 F-28A-1 (*n* = 1)	
34	34 (*n* = 1)	17A-34-1 (*n* = 1) ^b^	
unknown	unknown (*n* = 2)^d^	unknown (*n* = 2)	

^a^21 different sequetypes were identified

^b^Five isolates with ambiguous sequetype result, mPCR confirmed them

^c^Thirteen 23 F isolates were of new sequetypes after mPCR was performed

^d^Four isolates were untypeable by mPCR. Two isolates were identified by *cpsB* sequetyping. Two isolates were also unknown by sequencing

When the Blastn result was not a 100 % match with any GenBank sequences, the sequences (if with good sequencing quality for both directions) potentially represented new *cpsB* sequetypes. For example, four new 23 F *cpsB* sequetypes, confirmed by 23 F specific PCR were found among thirteen 23 F isolates (Additional file 4: Table S3). Among the 27 serogroup 6 isolates, the distribution of sequetypes was as follows; 6B-1 (1/27, 3.7 %), 6C-6D-1 (14/27, 51.8 %), 6B-6E-6X-1 (12/27, 44.4 %). Two isolates (ID 250, 268) produced *cpsB* amplicons but they were non-typeable by both *cpsB* sequetyping and sequential multiplex PCR (Additional file 4: Table S3).

### Sequential multiplex PCR results

After *cpsB* sequetyping results were known, isolates that presumptively belonged to relevant serotypes were further tested by multiplex PCR to confirm the results, resolve discrepant results, or identify those that shared the same *cpsB* sequetype (Table 1, Additional file 4: Table S3, and Fig. 1). Overall, 115 (59.5 %) of sequetyping results needed confirmation by selected mPCR to give a definite serotype. The level of agreement between *cpsB* sequetyping and multiplex PCR results was 92.2 %. Five isolates, for which sequencing gave ambiguous result as serotypes 15A-33B-1 (2 isolates), 13-20A-20B-1 (2 isolates), and 17A-34-1 (1 isolate), were confirmed by mPCR that they were serotypes 15 F/15A, 20 and 34, respectively. Thirteen serotype 23 F isolates belonging to four new *cpsB* sequetypes, were assigned new sequetype names after mPCR confirmed them as serotype 23 F. Four isolates identified by *cpsB* sequetyping as 10B, 28 F/28A, were non-typeable by mPCR because the serotype primer sets were not included in the USA CDC multiplex reaction scheme (Table 1, Additional file 4: Table S3). Two isolates (ID 250, 268 in Additional file 4: Table S3) showed unknown sequetype in *cpsB* sequetyping, and were also not amplified by any specific primer sets.

### Serotypes 6A-6D specific PCRs results

The distribution of serotypes 6A-6D among the 27 serogroup 6 isolates were: 6A (12/27, 44.4 %), 6B (13/27, 48.1 %) and 6C (2/27, 7.4 %) and serotype 6D was not detected.

### Serotype distribution

Using the sequence-based method selectively supplemented with sequential multiplex PCR and serotypes 6A-6D specific PCRs strategy, sixteen serotypes were identified from 193 *S. pneumoniae* isolates. They included 19 F (67, 34.7 %), 23 F (33, 17.1 %), 19A (23, 11.9 %), 14 (14, 7.3 %), 15B/15C (13, 6.7 %), 6B (13, 6.7 %), 6A (12, 6.2 %), 9 V/9A (3, 1.6 %); serotypes 6C, 3, 15 F/15A and 20 (2 each, 1.1 %); serotypes 10B, 28 F/28A and 34 (1 each, 0.5 %). The 10-valent PCV (PCV-10) vaccines cover 67.4 % of the serotypes identified, whilst the 13-valent PCV (PCV-13) covers 86.5 %. A total of 126 isolates were from patients less than 2 years of age, including 39 isolates of serotypes 19 F (30.9 %), 23 of 23 F (18.3 %), 16 of 19A (12.7 %), 10 of 15B/15C (7.9 %), 10 of 6A (7.9 %), 9 of 14 (7.1 %), 8 of 6B (6.3 %); 2 each of 20 and 15 F/15A (1.6 %); and 1 each of 9 V/9A, 3, 34, 10B and 23A (0.8 %). PCV-10 covers 70.6 % of these strains whilst PCV-13 covers 92 %. There was no significant difference between serotype distribution and clinical presentation (see Additional file 5: Table S4).

### Antimicrobial susceptibility

Susceptibility results for the *S. pneumoniae* isolates are shown in Table 2. The resistance rates for erythromycin, clindamycin, sulfamethoxazole-trimethoprim and tetracycline, ranged from 87.6 to 97.4 %. According to the revised CLSI breakpoints for parenteral penicillin, the prevalence rates for penicillin resistance were 1.0 and 88.6 % in the non-meningitis and meningitis isolates, respectively. The proportion of isolates resistant to ceftriaxone was 5.2 % for non-meningitis, and 25.4 % for meningitis isolates. All the isolates were susceptible to vancomycin. The percentage of MDR isolates was 86.8 % (167/193), and the most common pattern was resistance to erythromycin + clindamycin + sulfamethoxazole-trimethoprim (167/193, 86.8 %), followed by resistance to erythromycin + clindamycin + sulfamethoxazole-trimethoprim + tetracycline (150/193, 77.9 %), and erythromycin + clindamycin + sulfamethoxazole-trimethoprim + tetracycline + chloramphenicol (22/193, 11.5 %). Antibiotic resistance was clustered mainly in serotype 19 F, with resistant rates to parenteral penicillin, ceftriaxone and erythromycin of 1.5, 14.9, and 97 % respectively. The other half of the penicillin resistant isolates was identified as serotype 23 F (3 %) (Additional file 6: Table S5). For the penicillin, ceftriaxone parenteral resistant non-meningitis isolates, the multidrug resistance patterns were; erythromycin + clindamycin + sulfamethoxazole-trimethoprim + tetracycline + chloramphenicol + penicillin + ceftriaxone (*n* = 1); erythromycin + clindamycin + sulfamethoxazole-trimethoprim + tetracycline + penicillin + ceftriaxone (*n* = 1); erythromycin + clindamycin + sulfamethoxazole-trimethoprim + tetracycline + ceftriaxone (*n* =6); erythromycin + clindamycin + sulfamethoxazole-trimethoprim + ceftriaxone (*n* =2).

**Table 2:** Prevalence of antibiotic susceptibility to nine antimicrobials for 193 *S. pneumoniae* isolates from children

Antimicrobial	No. (%) of isolates			MIC (ug/mL)		
	Susceptible	Intermediate	Resistant	MIC_50_	MIC_90_	Range
Penicillin						0.016–8.0
Non-meningitis isolates						
Parenteral	176 (91.2)	15 (7.8)	2 (1.0)	1	2	
Oral	22 (11.4)	95 (49.2)	76 (39.4)	1	2	
Meningitis isolates	22 (11.4)	0 (0)	171 (88.6)	1	2	
Ceftriaxone						0.016–6.0
Non-meningitis isolates	144 (74.6)	39 (20.2)	10 (5.2)	0.75	2	
Meningitis isolates	83 (43.0)	61 (31.6)	49 (25.4)	0.75	2	
Erythromycin	2 (1.0)	3 (1.6)	188 (97.4)			
Vancomycin	193 (100)	0(0)	0(0)			
Levofloxacin	191 (99)	2 (1.0)	0 (0)			
Tetracycline	9 (4.7)	12 (6.2)	172 (89.1)			
Chloramphenicol	168 (87.0)	0 (0)	25 (13)			
Sulfamethoxazole-trimethoprim	14 (7.3)	10 (5.2)	169 (87.6)			
Clindamycin	5 (2.6)	1 (0.5)	187 (96.9)			

## Discussion

Most DNA-based methods allow the identification of a limited number of *S. pneumoniae* serotypes or serogroups. Since not all of the 95 described capsular types cause serious infections, it is important to develop a capsular typing scheme targeting serotypes most frequently associated with serious diseases [11]. In addition, after introduction of the pneumococcal conjugate vaccines, serotyping or serotype prediction assays are needed to monitor serotype switch from vaccine serotypes to non-vaccine serotypes [21].

Because of the existence of 95 different *S. pneumoniae* capsular types, it is difficult to develop a simple practical molecular typing scheme based on genetic approaches. In the present study, we developed a strategy to address this challenge. To our knowledge, it is the most comprehensive *cpsB* sequetyping database to date. Having more serotype sequetypes and sequetypes with multiple identical *cpsB* sequences in the sequetyping database, leads to more accurate serotype prediction compared to Leung’s study.

For most of our local isolates (except 2 non-serotypeable), *cpsB* sequetyping would be a more straightforward way to predict serotypes. Although molecular assays are generally considered unaffordable for most developing countries, PCR reagents are commonly available in most laboratories in China, and are relatively inexpensive. Furthermore, commercial sequencing is also affordable, convenient and cheap (~U$ 2.5/each reaction) for the majority of clinical labs. In our laboratory, *cpsB* sequencing is performed when a sufficient number of samples have been submitted for a run, which makes the cost very reasonable, and enables the lab to operate more efficiently. However, we found that many GenBank sequences share the same *cpsB* sequences (Additional file 3: Table S2), between both related serotypes (antigenic cross-reaction) and unrelated serotypes (no antigenic cross-reaction), probably due to recombination events [22]. In this study, sequetyping characterized 34.2 % isolates to serotype level and 55.4 % isolates to serogroup level. Multiplex PCR (mPCR) was needed to make a definite serotype prediction or increase the serotype prediction accuracy. But in most cases, *cpsB* sequetyping had already defined the test isolates to a smaller serotype group range, which made mPCR set selection much easier and saved from having to perform eight sequential mPCR. We only needed to resolve those *cpsB* sequetypes which were shared by different serotypes (e.g., 13-20A-20B, 15A-33B, 17A-34) or for isolates non-typeable by *cpsB* sequetyping (Fig. 1). In fact, only 115 (59.5 %) of sequetyping results needed to be confirmed by selected sets of mPCR in this study.

Furthermore, using the mPCR, most serotypes were identified in the first three sets of mPCR reactions, and only seven (3.6 %) isolates required further testing of up to eight sequential sets of multiplex reactions. After we update our new *cpsB* sequetyping database in the future, the number of isolates requiring mPCR confirmation would be much less than 59.5 % isolates, with most of them identified in the first three mPCR reactions. We also identified some new sequetypes within serotypes 23 F, 6A, 15 F/15A, 3 and rare serotype 20, which will improve the serotype prediction accuracy besides reducing the necessity of performing eight sequential multiplex reactions.

Although molecular methods are becoming increasingly utilized for pneumococcal typing, phenotypic methods including Quellung reaction and latex agglutination, remain the most reliable way to discover possible false-positive PCR results, which is fairly rare, but can occur (Fig. 1) [23]. However, some isolates are genotypeable by microarray or sequencing, but non-typeable by the Quellung reaction and latex agglutination [10, 24], suggesting caution must be exercised when interpreting controversial results (Fig. 1). In reality, genotyping, no matter how accurate it is, is only a method for prediction of serotypes/serogroups, not a replacement method for conventional serotyping, because “serotype” is traditionally a phenotypic rather than a genotype based definition.

The distribution of *S. pneumoniae* serotypes differ by geographic region*.* Several studies conducted over the years in China demonstrated great diversity in the distribution of *S. pneumoniae* serotypes by region [25, 26]*.* Bao’an district, the biggest administrative region in Shenzhen City, has a population of 6 million, of which 90 % are floating (temporary) residents as this city shares a border with Hong Kong. In this region, the immunization level for PCV7 vaccine has been less than 1 % for the past 5 years. Our study identified 16 serotypes, seven (19 F, 23 F, 19A, 14, 15B/15C, 6B and 6A) of which accounted for 90.7 % of the isolates, which is in general agreement to another study in Shenzhen [3]. Furthermore, our study confirmed previous findings showing that serotype 19A is one of the most common serotypes in Shenzhen [3] and China [27], which is not related to the introduction of PCV-7 vaccine, but to widespread antimicrobial use, similar to the situation in Korea [28]. A further 15 (7.8 %) of the 193 isolates belonged to serogroup 15 (2 serotype 15 F/15A isolates; 13 serotype 15B/15C isolates), which is quite similar to the pre-PVC7 period proportion in Hong Kong for serogroup 15 (5.7 %) [29].

It has been reported that serotypes 1, 2, 7, 9, 14, and 16 are among the most invasive serotypes, whilst serotypes 3, 6, 15, 19, and 23 are considered least invasive serotypes [30, 31]. However, Picazo et al. reported that serotype 19A was linked to non-respiratory IPDs in children of <24 months [32], whilst Hausdorff et al. found that serotypes 1 and 14 were more often isolated from blood, and serogroups 3, 19, and 23, more often isolated from middle ear fluid [33]. In the present study, no significant differences were noted between serotype and clinical presentation, which could be due to limited number of isolates and/or disease categories studied.

Prevention of pneumococcal disease includes vaccination with pneumococcal polysaccharide conjugate vaccines especially in children. Based on the distribution of serotypes in all patients in our study population (age from 27 days to 6 years and 5 months), the PCV-10 vaccine would cover 67.4 % of the serotypes, whilst the PCV-13 vaccine would cover 86.5 % of the serotypes found in the area. The major target population for vaccination and prevention is children under 2 years of age. PCV-13 still showed higher coverage (92 %) in <2 year old patients in our study population compare to PCV-10 (70.6 %). Specifically, the significantly increased coverage by PCV13 in our study population is due to high prevalence of serotypes 19A (23, 11.9 %) and 6A (12, 6.2 %), which together with serotype 3, are contained in the PCV-13 vaccine but not in the PCV-10 [3]. These findings suggest that PCV-13 should be the major target for future development and applications in our community, since PCV-7 is no longer available, and PCV-10 has less coverage.

The low prevalence rate of parenteral penicillin resistance (1.0 %) among the non-meningitis isolates in our study is noted, and suggests that hospitalized children with non-meningitis pneumococcal infections can be treated with parenteral penicillin. It has been reported that seven (6A, 6B, 9 V, 14, 19A, 19 F, 23 F) out of 95 serotypes are associated with antibiotic resistance [34]. Interestingly, these seven serotypes accounted for 85.5 % of our 193 isolates, which could explain the high prevalence rate of multidrug resistance (86.8 %) in the present study (Additional file 6: Table S5).

This study has several limitations. First, the number of isolates from blood and cerebrospinal fluid was small, which limited statistical power. Furthermore, 87.6 % isolates were from sputum, some of which may be colonizing organisms since certain *S. pneumoniae* serotypes have a propensity for colonization without necessarily causing disease. However, it is believed that invasive disease originates from colonization and that serotype distribution among colonizing strains, is an indicator of the diversity of pneumococcal strains circulating in the community [35]. Furthermore, our sputum specimen sampling strategy, including using the quality criteria, would have minimized the level of contamination from colonizing strains. Secondly, all data presented here is from one of the biggest district hospitals, so we can’t overplay the data to represent more rural regions though it would be representative of our Bao’an District hospitalized children.

## Conclusions

This study provides a cost-effective alternative *S. pneumoniae* serotype prediction strategy to conventional serotyping. We showcased the utility of this new serotyping strategy by identifying serotypes of 193 *S. pneumoniae* isolates from children. This strategy enables most routine laboratories equipped with PCR to predict the majority of pneumococcal serotypes without the need for an expensive set of serological reagents in China. This study confirms that serotype 19A is common in China, and that PCV13 vaccine would be important for future vaccination in areas such as Shenzhen. Considering the low resistance rate in non-meningitis isolates to parenteral penicillin, clinicians should be encouraged to increase the use of penicillin to treat penicillin-susceptible non-meningitis pneumococcal infections, instead of using broader-spectrum antimicrobials. Continued surveillance of the serotype distribution and antimicrobial susceptibility of *S. pneumoniae* isolates in China is warranted.

### Availability of data and materials

The datasets supporting the conclusions of this article are included within the article and its additional files. Patient age and gender were part of the original dataset used in our study. We have, however, removed this information from the clinical dataset provided in Additional file 1: Table S1 in order to protect the patients’ identity. The GenBank/EMBL/DDBJ accession numbers for the new *cpsB* sequences from this study have been submitted to GenBank with accession numbers KT164777-KT164783 and are listed in Additional file 4: Table S3.

## Additional files

Additional file 1: Table S1. Clinical information of our 193 isolates. (DOC 318 kb) Additional file 2: Figure S1. The algorithm show how we build up the comprehensive GenBank *cpsB* seuquetyping database as Additional file 3: Table S2. (TIFF 19 kb) Additional file 3: Table S2. Comprehensive *cpsB* sequetyping database based on GenBank sequences (until Jan 1, 2015). (DOCX 47 kb) Additional file 4: Table S3. The *cpsB* sequetyping results of our 193 local isolates. (DOCX 40 kb) Additional file 5: Table S4. Serotype distribution by clinical presentation among our 193 *S. pneumonie* isolates from children. (DOC 44 kb) Additional file 6: Table S5. Percentages of resistant to antibiotics for serotypes with 10 or more isolates. (DOC 36 kb)

## Abbreviations

CDC: centers for disease control and prevention
CLSI: the Clinical and Laboratory Standards Institute
MDR: multidrug- resistance
mPCR: multiplex PCR
PCV-10: 10-valent PCV
PCV-13: 13-valent PCV
PCV7: 7-valent pneumococcal conjugate vaccine
*S. pneumoniae*: *Streptococcus pneumoniae*
