# Supplementary material for: Using a practical molecular capsular serotype prediction strategy to investigate Streptococcus pneumoniae serotype distribution and antimicrobial resistance in Chinese local hospitalized children
Source: BMC Pediatr. 2016 Apr 26;16:53. doi: 10.1186/s12887-016-0589-7 (PMC4847217; doi:10.1186/s12887-016-0589-7)
Supplement: Additional file 1: Table S1. — Clinical information of our 193 isolates. (DOC 318 kb) [file 12887_2016_589_MOESM1_ESM.doc]

**Additional file 1: Table S1. Clinical information of our 193 isolates.**

| **Strain**  **ID no.** | **Serotype** | **Specimens**  **source** | **Collection**  **Date**^a^ | **Clinical Diagnosis** |
| --- | --- | --- | --- | --- |
| 230 | 3 | sputum | 2013-11-03 | non-severe pneumonia |
| 282 | 3 | sputum | 2013-11-08 | non-severe pneumonia |
| 288 | 6B | sputum | 2013-11-13 | non-severe pneumonia |
| 21 | 6B | blood | 2010-03-07 | primary bacteremia |
| 40 | 6B | sputum | 2012-05-07 | non-severe pneumonia |
| 52 | 6B | sputum | 2012-05-08 | non-severe pneumonia |
| 69 | 6B | sputum | 2012-04-26 | non-severe pneumonia |
| 75 | 6B | sputum | 2012-03-06 | non-severe pneumonia |
| 105 | 6B | sputum | 2012-02-11 | non-severe pneumonia |
| 109 | 6B | sputum | 2012-02-20 | non-severe pneumonia |
| 281 | 6B | sputum | 2013-10-07 | non-severe pneumonia |
| 286 | 6B | sputum | 2013-11-16 | non-severe pneumonia |
| 297 | 6B | sputum | 2013-12-18 | non-severe pneumonia |
| 299 | 6B | sputum | 2013-12-24 | non-severe pneumonia |
| 313 | 6B | sputum | 2013-11-21 | non-severe pneumonia |
| 42 | 6A | sputum | 2012-05-08 | non-severe pneumonia |
| 73 | 6A | sputum | 2012-03-06 | non-severe pneumonia |
| 97 | 6A | sputum | 2012-03-11 | severe pneumonia |
| 99 | 6A | sputum | 2012-03-15 | non-severe pneumonia |
| 100 | 6A | sputum | 2012-03-09 | non-severe pneumonia |
| 220 | 6A | sputum | 2013-03-19 | severe pneumonia |
| 240 | 6A | sputum | 2013-03--15 | non-severe pneumonia |
| 242 | 6A | sputum | 2013-04--14 | severe pneumonia |
| 244 | 6A | sputum | 2013-04-18 | non-severe pneumonia |
| 267 | 6A | sputum | 2013-07-25 | non-severe pneumonia |
| 276 | 6A | sputum | 2013-09-23 | non-severe pneumonia |
| 148 | 6A | cerebrospinal fluid | 2013-09-22 | meningitis |
| 219 | 6C | pus | 2013-06-04 | urinary tract infections |
| 307 | 6C | pleural fluid | 2013-09-13 | severe pneumonia |
| 238 | 9V/9A | blood | 2013-06-14 | primary bacteremia |
| 298 | 9V/9A | sputum | 2013-12-02 | severe pneumonia |
| 308 | 9V/9A | blood | 2013-12-16 | primary bacteremia |
| 231 | 10B | sputum | 2013-11-28 | non-severe pneumonia |
| 19 | 14 | blood | 2009-11-21 | primary bacteremia |
| 300 | 14 | sputum | 2012-03-07 | non-severe pneumonia |
| 83 | 14 | sputum | 2011-12-29 | non-severe pneumonia |
| 91 | 14 | sputum | 2012-02-28 | non-severe pneumonia |
| 136 | 14 | sputum | 2012-03-10 | non-severe pneumonia |
| 137 | 14 | blood | 2012-06-26 | primary bacteremia |
| 138 | 14 | sputum | 2013-11-06 | non-severe pneumonia |
| 229 | 14 | sputum | 2013-09-23 | severe pneumonia |
| 277 | 14 | sputum | 2013-12-26 | severe pneumonia |
| 295 | 14 | sputum | 2013-12-14 | non-severe pneumonia |
| 301 | 14 | sputum | 2013-12-19 | non-severe pneumonia |
| 306 | 14 | blood | 2013-10-22 | primary bacteremia |
| 309 | 14 | blood | 2012-12-25 | primary bacteremia |
| 310 | 14 | cerebrospinal fluid | 2012-12-13 | meningitis |
| 214 | 15A/15F | sputum | 2013-03-03 | non-severe pneumonia |
| 293 | 15A/15F | sputum | 2013-12-18 | non-severe pneumonia |
| 72 | 15B/15C | sputum | 2012-02-07 | non-severe pneumonia |
| 87 | 15B/15C | sputum | 2012-03-23 | severe pneumonia |
| 107 | 15B/15C | sputum | 2012-02-11 | non-severe pneumonia |
| 256 | 15B/15C | sputum | 2013-05-14 | severe pneumonia |
| 259 | 15B/15C | sputum | 2013-06-18 | non-severe pneumonia |
| 312 | 15B/15C | sputum | 2013-12-11 | non-severe pneumonia |
| 29 | 15B/15C | sputum | 2012-05-21 | non-severe pneumonia |
| 34 | 15B/15C | sputum | 2012-05-17 | non-severe pneumonia |
| 113 | 15B/15C | sputum | 2012-03-12 | non-severe pneumonia |
| 123 | 15B/15C | sputum | 2012-02-04 | non-severe pneumonia |
| 213 | 15B/15C | sputum | 2013-03-03 | non-severe pneumonia |
| 215 | 15B/15C | sputum | 2013-03-04 | non-severe pneumonia |
| 216 | 15B/15C | sputum | 2013-03-20 | non-severe pneumonia |
| 17 | 19A | blood | 2009-01-07 | primary bacteremia |
| 38 | 19A | sputum | 2012-05-20 | severe pneumonia |
| 49 | 19A | sputum | 2012-05-07 | non-severe pneumonia |
| 64 | 19A | sputum | 2012-04-20 | non-severe pneumonia |
| 65 | 19A | sputum | 2012-04-20 | non-severe pneumonia |
| 74 | 19A | sputum | 2012-02-07 | non-severe pneumonia |
| 90 | 19A | sputum | 2012-03-09 | non-severe pneumonia |
| 93 | 19A | sputum | 2012-03-05 | non-severe pneumonia |
| 101 | 19A | sputum | 2012-03-02 | severe pneumonia |
| 129 | 19A | sputum | 2012-03-13 | severe pneumonia |
| 131 | 19A | sputum | 2012-02-12 | non-severe pneumonia |
| 217 | 19A | blood | 2013-03-14 | primary bacteremia |
| 218 | 19A | sputum | 2013-09-18 | non-severe pneumonia |
| 245 | 19A | sputum | 2013-04-25 | non-severe pneumonia |
| 253 | 19A | sputum | 2013-05-01 | non-severe pneumonia |
| 272 | 19A | sputum | 2013-09-06 | severe pneumonia |
| 275 | 19A | sputum | 2013-09-28 | non-severe pneumonia |
| 278 | 19A | sputum | 2013-09-29 | non-severe pneumonia |
| 280 | 19A | sputum | 2013-10-25 | non-severe pneumonia |
| 283 | 19A | sputum | 2013-11-24 | non-severe pneumonia |
| 285 | 19A | sputum | 2013-11-19 | severe pneumonia |
| 290 | 19A | sputum | 2013-12-13 | non-severe pneumonia |
| 311 | 19A | sputum | 2013-11-16 | severe pneumonia |
| 5 | 19F | sputum | 2011-04-12 | severe pneumonia |
| 6 | 19F | sputum | 2012-04-19 | non-severe pneumonia |
| 9 | 19F | sputum | 2012-04-19 | non-severe pneumonia |
| 20 | 19F | blood | 2009-01-16 | primary bacteremia |
| 22 | 19F | blood | 2010-04-03 | primary bacteremia |
| 23 | 19F | blood | 2011-03-02 | primary bacteremia |
| 24 | 19F | blood | 2010-11-12 | primary bacteremia |
| 25 | 19F | sputum | 2013-02-15 | non-severe pneumonia |
| 26 | 19F | sputum | 2011-06-24 | non-severe pneumonia |
| 31 | 19F | sputum | 2012-05-15 | non-severe pneumonia |
| 43 | 19F | sputum | 2012-05-10 | non-severe pneumonia |
| 54 | 19F | sputum | 2012-05-09 | severe pneumonia |
| 56 | 19F | sputum | 2012-04-25 | non-severe pneumonia |
| 58 | 19F | sputum | 2012-04-26 | non-severe pneumonia |
| 61 | 19F | sputum | 2013-05-10 | non-severe pneumonia |
| 66 | 19F | sputum | 2011-03-19 | non-severe pneumonia |
| 67 | 19F | sputum | 2012-04-22 | severe pneumonia |
| 68 | 19F | sputum | 2012-04-29 | non-severe pneumonia |
| 76 | 19F | sputum | 2012-03-04 | non-severe pneumonia |
| 82 | 19F | sputum | 2012-03-01 | severe pneumonia |
| 86 | 19F | sputum | 2012-02-29 | non-severe pneumonia |
| 88 | 19F | sputum | 2012-03-06 | severe pneumonia |
| 94 | 19F | sputum | 2012-02-12 | non-severe pneumonia |
| 124 | 19F | sputum | 2012-02-12 | severe pneumonia |
| 289 | 19F | sputum | 2013-12-13 | non-severe pneumonia |
| 292 | 19F | sputum | 2013-12-13 | severe pneumonia |
| 106 | 19F | sputum | 2012-02-07 | non-severe pneumonia |
| 112 | 19F | sputum | 2012-03-03 | non-severe pneumonia |
| 115 | 19F | sputum | 2012-02-05 | non-severe pneumonia |
| 117 | 19F | sputum | 2012-02-09 | non-severe pneumonia |
| 120 | 19F | sputum | 2012-02-20 | non-severe pneumonia |
| 122 | 19F | sputum | 2012-02-08 | severe pneumonia |
| 125 | 19F | sputum | 2012-02-06 | severe pneumonia |
| 126 | 19F | sputum | 2012-02-15 | non-severe pneumonia |
| 133 | 19F | sputum | 2012-02-06 | severe pneumonia |
| 211 | 19F | sputum | 2013-03-02 | non-severe pneumonia |
| 222 | 19F | sputum | 2013-06-14 | non-severe pneumonia |
| 223 | 19F | sputum | 2013-02-18 | non-severe pneumonia |
| 224 | 19F | sputum | 2013-02-25 | non-severe pneumonia |
| 225 | 19F | sputum | 2013-02-26 | non-severe pneumonia |
| 227 | 19F | sputum | 2013-11-24 | non-severe pneumonia |
| 232 | 19F | pus | 2013-03-04 | cellulitis |
| 235 | 19F | sputum | 2013-11-16 | non-severe pneumonia |
| 239 | 19F | sputum | 2013-03-12 | non-severe pneumonia |
| 241 | 19F | sputum | 2013-02-11 | non-severe pneumonia |
| 243 | 19F | sputum | 2013-05-23 | severe pneumonia |
| 247 | 19F | sputum | 2013-04-13 | non-severe pneumonia |
| 248 | 19F | sputum | 2013-05-25 | non-severe pneumonia |
| 249 | 19F | sputum | 2013-06-29 | non-severe pneumonia |
| 251 | 19F | sputum | 2013-04-13 | non-severe pneumonia |
| 252 | 19F | sputum | 2013-05-12 | non-severe pneumonia |
| 254 | 19F | sputum | 2013-05-10 | non-severe pneumonia |
| 257 | 19F | sputum | 2013-05-14 | non-severe pneumonia |
| 258 | 19F | sputum | 2013-06-23 | severe pneumonia |
| 260 | 19F | sputum | 2013-06-28 | non-severe pneumonia |
| 262 | 19F | sputum | 2013-07-29 | severe pneumonia |
| 265 | 19F | sputum | 2013-06-06 | non-severe pneumonia |
| 269 | 19F | sputum | 2013-09-15 | non-severe pneumonia |
| 270 | 19F | sputum | 2013-08-26 | severe pneumonia |
| 271 | 19F | sputum | 2013-08-22 | non-severe pneumonia |
| 273 | 19F | sputum | 2013-09-23 | non-severe pneumonia |
| 284 | 19F | sputum | 2013-10-13 | non-severe pneumonia |
| 287 | 19F | sputum | 2013-11-16 | non-severe pneumonia |
| 291 | 19F | sputum | 2013-10-18 | non-severe pneumonia |
| 302 | 19F | sputum | 2013-12-13 | non-severe pneumonia |
| 304 | 19F | sputum | 2013-12-27 | non-severe pneumonia |
| 305 | 19F | blood | 2013-09-26 | primary bacteremia |
| 234 | 20 | sputum | 2013-03-28 | severe pneumonia |
| 255 | 20 | sputum | 2013-05-20 | non-severe pneumonia |
| 266 | 23A | sputum | 2013-07-13 | non-severe pneumonia |
| 314 | 23A | sputum | 2013-12-21 | non-severe pneumonia |
| 10 | 23F | sputum | 2012-04-02 | non-severe pneumonia |
| 45 | 23F | sputum | 2012-05-13 | non-severe pneumonia |
| 77 | 23F | sputum | 2012-02-27 | non-severe pneumonia |
| 81 | 23F | sputum | 2012-03-09 | severe pneumonia |
| 80 | 23F | sputum | 2012-02-28 | non-severe pneumonia |
| 103 | 23F | sputum | 2012-03-02 | severe pneumonia |
| 104 | 23F | sputum | 2012-02-29 | severe pneumonia |
| 108 | 23F | sputum | 2012-02-07 | non-severe pneumonia |
| 118 | 23F | sputum | 2012-02-12 | non-severe pneumonia |
| 128 | 23F | sputum | 2012-03-20 | non-severe pneumonia |
| 212 | 23F | sputum | 2013-03-03 | non-severe pneumonia |
| 226 | 23F | sputum | 2013-02-13 | non-severe pneumonia |
| 228 | 23F | sputum | 2013-11-08 | severe pneumonia |
| 237 | 23F | sputum | 2013-09-19 | non-severe pneumonia |
| 246 | 23F | sputum | 2013-04-27 | non-severe pneumonia |
| 274 | 23F | sputum | 2013-09-08 | severe pneumonia |
| 294 | 23F | sputum | 2013-12-17 | non-severe pneumonia |
| 141 | 23F | pleural fluid | 2013-08-12 | severe pneumonia |
| 142 | 23F | pleural fluid | 2013-12-21 | severe pneumonia |
| 149 | 23F | blood | 2013-06-06 | primary bacteremia |
| 16 | 23F | sputum | 2012-04-12 | non-severe pneumonia |
| 35 | 23F | sputum | 2012-05-16 | non-severe pneumonia |
| 37 | 23F | sputum | 2012-05-18 | non-severe pneumonia |
| 135 | 23F | sputum | 2012-02-20 | non-severe pneumonia |
| 221 | 23F | sputum | 2013-01-05 | non-severe pneumonia |
| 261 | 23F | sputum | 2013-06-04 | non-severe pneumonia |
| 263 | 23F | sputum | 2013-06-18 | non-severe pneumonia |
| 264 | 23F | blood | 2013-02-14 | primary bacteremia |
| 279 | 23F | sputum | 2013-10-17 | non-severe pneumonia |
| 32 | 23F | sputum | 2012-05-16 | non-severe pneumonia |
| 233 | 23F | sputum | 2013-11-14 | severe pneumonia |
| 296 | 23F | sputum | 2013-12-12 | non-severe pneumonia |
| 144 | 23F | blood | 2013-10-12 | primary bacteremia |
| 78 | 28F/28A | sputum | 2012-03-07 | non-severe pneumonia |
| 236 | 34 | sputum | 2013-11-23 | non-severe pneumonia |
| 250 | unknown | sputum | 2013-04-19 | non-severe pneumonia |
| 268 | unknown | sputum | 2013-12-10 | non-severe pneumonia |

Notes:

^a^year-month-date.
