# Supplementary material for: Using a practical molecular capsular serotype prediction strategy to investigate Streptococcus pneumoniae serotype distribution and antimicrobial resistance in Chinese local hospitalized children
Source: BMC Pediatr. 2016 Apr 26;16:53. doi: 10.1186/s12887-016-0589-7 (PMC4847217; doi:10.1186/s12887-016-0589-7)
Supplement: Additional file 3: Table S2. — Comprehensive cpsB sequetyping database based on GenBank sequences (until Jan 1, 2015). (DOCX 47 kb) [file 12887_2016_589_MOESM3_ESM.docx]

**Additional file 3: Table S2 .Comprehensive *cpsB* sequetyping database based on GenBank sequences (until Jan 1, 2015).**

| GenBank accession  numbers | Serotypes | Reference *cpsB* GenBank sequence (732-bp *cpsB* positions on the selected reference sequences)* | Sequetype  (with identical *cpsB* GenBank sequence numbers) |
| --- | --- | --- | --- |
| CR931632 | 1 | CR931632 (4508..5239) | 1-1 (15) |
| CP000920 | 1 |  | 1-1 (15) |
| CP001845 | 1 |  | 1-1 (15) |
| FQ312030 | 1 |  | 1-1 (15) |
| FQ312039 | 1 |  | 1-1 (15) |
| FQ312042 | 1 |  | 1-1 (15) |
| JN642309 | 1 |  | 1-1 (15) |
| JN660116 | 1 |  | 1-1 (15) |
| JN660117 | 1 |  | 1-1 (15) |
| JN660118 | 1 |  | 1-1 (15) |
| JN660119 | 1 |  | 1-1 (15) |
| JN660120 | 1 |  | 1-1 (15) |
| JN660121 | 1 |  | 1-1 (15) |
| JN660122 | 1 |  | 1-1 (15) |
| Z83335 | 1 |  | 1-1 (15) |
| CR931633 | 2 | CR931633 (3109..3840)/CR931713 (3031..3742) | 2-41A-1 (3) |
| AF026471 | 2 |  | 2-41A-1 (3) |
| CP000410 | 2 |  | 2-41A-1 (3) |
| JN660123 | 2 | JN660123 (1..732) | 2-1 (1) |
| CR931634 | 3 | CR931634 (2750..3480) | 3-1 (1) |
| HE983624 | 3 | HE983624 (1724948..1724218) | 3-2 (12) |
| FQ312027 | 3 |  | 3-2 (12) |
| FQ312041 | 3 |  | 3-2 (12) |
| FQ312043 | 3 |  | 3-2 (12) |
| FQ312044 | 3 |  | 3-2 (12) |
| FQ312045 | 3 |  | 3-2 (12) |
| JN680145 | 3 |  | 3-2 (12) |
| JQ743514 | 3 |  | 3-2 (12) |
| JQ743515 | 3 |  | 3-2 (12) |
| JQ743516 | 3 |  | 3-2 (12) |
| JQ743517 | 3 |  | 3-2 (12) |
| JQ743518 | 3 |  | 3-2 (12) |
| Z47210 | 3 | Z47210 (2910..3640) | 3-3 (1) |
| JN660145 | 3 | JN660145 (1..732) | 3-4 (1) |
| CR931635 | 4 | CR931635 (2750..3480) | 4-1 (12) |
| AE005672 | 4 |  | 4-1 (12) |
| AF316639 | 4 |  | 4-1 (12) |
| JN660124 | 4 |  | 4-1 (12) |
| JN660125 | 4 |  | 4-1 (12) |
| JN664256 | 4 |  | 4-1 (12) |
| JN680110 | 4 |  | 4-1 (12) |
| JN680122 | 4 |  | 4-1 (12) |
| JN680123 | 4 |  | 4-1 (12) |
| JQ743519 | 4 |  | 4-1 (12) |
| JQ743520 | 4 |  | 4-1 (12) |
| JQ743521 | 4 |  | 4-1 (12) |
| CR931637 | 5 | CR931637 (1424..2155) | 5-1 (3) |
| AY336008 | 5 |  | 5-1 (3) |
| JQ743525 | 5 |  | 5-1 (3) |
| CP000918 | 5 | CP000918 (365377..366108) | 5-2 (5) |
| JN660126 | 5 |  | 5-2 (5) |
| JQ743522 | 5 |  | 5-2 (5) |
| JQ743523 | 5 |  | 5-2 (5) |
| JQ743524 | 5 |  | 5-2 (5) |
| JF911488 | 6A | JF911488 (1448..2179) | 6A-1 (7) |
| JF911490 | 6A |  | 6A-1 (7) |
| JF911493 | 6A |  | 6A-1 (7) |
| JF911499 | 6A |  | 6A-1 (7) |
| JF911505 | 6A |  | 6A-1 (7) |
| JQ743526 | 6A |  | 6A-1 (7) |
| JQ743529 | 6A |  | 6A-1 (7) |
| JF911487 | 6A | JF911487 (1448..2179) | 6A-2 (6) |
| JF911491 | 6A |  | 6A-2 (6) |
| JF911495 | 6A |  | 6A-2 (6) |
| JF911496 | 6A |  | 6A-2 (6) |
| JQ743527 | 6A |  | 6A-2 (6) |
| JQ743528 | 6A |  | 6A-2 (6) |
| AY078347 | 6A | AY078347 (1666..3397) | 6A-3 (1) |
| JN680147 | 6A | JN680147 (1..732) | 6A-4 (1) |
| JF911489 | 6A | AF316640 (2651..3382)/JF911489 (1448..2179)/KC832411 (2603..3334) | 6A-6B-6F-1 (15) |
| JF911497 | 6A |  | 6A-6B-6F-1 (15) |
| CR931638 | 6A | CR931638 (2756..3487)/KC832410 (2607..3338) | 6A-6E-1 (11) |
| JF911492 | 6A |  | 6A-6E-1 (11) |
| JF911494 | 6A |  | 6A-6E-1 (11) |
| JN660127 | 6A |  | 6A-6E-1 (11) |
| JN680106 | 6A |  | 6A-6E-1 (11) |
| JN680118 | 6A |  | 6A-6E-1 (11) |
| JN680128 | 6A |  | 6A-6E-1 (11) |
| JN680138 | 6A |  | 6A-6E-1 (11) |
| JN680146 | 6A |  | 6A-6E-1 (11) |
| JQ743530 | 6A |  | 6A-6E-1 (11) |
| CR931639 | 6B | CR931639 (2654..3385) | 6B-1 (1) |
| JN642311 | 6B | JN642311 (1..732) | 6B-2 (1) |
| JN642314 | 6B | JN642314 (1..732) | 6B-3 (1) |
| KC522500 | 6B | KC522500 (1..732) | 6B-4 (1) |
| KC522501 | 6B | KC522501 (1..732) | 6B-5 (1) |
| KC522502 | 6B | KC522502 (1..732) | 6B-6 (1) |
| AF316640 | 6B | AF316640 (2651..3382)/JF911489 (1448..2179)/KC832411 (2603..3334) | 6A-6B-6F-1 (15) |
| JF911498 | 6B |  | 6A-6B-6F-1 (15) |
| JF911500 | 6B |  | 6A-6B-6F-1 (15) |
| JF911501 | 6B |  | 6A-6B-6F-1 (15) |
| JF911502 | 6B |  | 6A-6B-6F-1 (15) |
| JF911503 | 6B |  | 6A-6B-6F-1 (15) |
| JN660128 | 6B |  | 6A-6B-6F-1 (15) |
| JN660129 | 6B |  | 6A-6B-6F-1 (15) |
| JN680105 | 6B |  | 6A-6B-6F-1 (15) |
| JN680136 | 6B |  | 6A-6B-6F-1 (15) |
| JN680137 | 6B |  | 6A-6B-6F-1 (15) |
| JN680142 | 6B |  | 6A-6B-6F-1 (15) |
| AF246897 | 6B | AF246897 (1666..2397)/KM114241 (1..732)/KC522494 (1..732) | 6B-6E-6X-1 (18) |
| CP002176 | 6B |  | 6B-6E-6X-1 (18) |
| JF911504 | 6B |  | 6B-6E-6X-1 (18) |
| JF911507 | 6B |  | 6B-6E-6X-1 (18) |
| JN642310 | 6B |  | 6B-6E-6X-1 (18) |
| JN642312 | 6B |  | 6B-6E-6X-1 (18) |
| JN642313 | 6B |  | 6B-6E-6X-1 (18) |
| JN642315 | 6B |  | 6B-6E-6X-1 (18) |
| JN680143 | 6B |  | 6B-6E-6X-1 (18) |
| JN680148 | 6B |  | 6B-6E-6X-1 (18) |
| EF538714 | 6C | EF538714 (3002..3733)/HM171374 (4356..5087) | 6C-6D-1 (7) |
| HM448897 | 6C |  | 6C-6D-1 (7) |
| JF911509 | 6C |  | 6C-6D-1 (7) |
| JF911510 | 6C |  | 6C-6D-1 (7) |
| JF911515 | 6C |  | 6C-6D-1 (7) |
| JN660130 | 6C |  | 6C-6D-1 (7) |
| HM171374 | 6D | EF538714 (3002..3733)/HM171374 (4356..5087) | 6C-6D-1 (7) |
| KM114241 | 6E | AF246897 (1666..2397)/KM114241 (1..732)/KC522494 (1..732) | 6B-6E-6X-1 (18) |
| KM114229 | 6E |  | 6B-6E-6X-1 (18) |
| KM114235 | 6E |  | 6B-6E-6X-1 (18) |
| KC832410 | 6E | CR931638 (2756..3487)/KC832410 (2607..3338) | 6A-6E-1 (11) |
| KC832411 | 6F | AF316640 (2651..3382)/JF911489 (1448..2179)/KC832411 (2603..3334) | 6A-6B-6F-1 (15) |
| KC522494 | 6X | AF246897 (1666..2397)/KM114241 (1..732)/KC522494 (1..732) | 6B-6E-6X-1 (18) |
| KC522495 | 6X |  | 6B-6E-6X-1 (18) |
| KC522496 | 6X |  | 6B-6E-6X-1 (18) |
| KC522497 | 6X |  | 6B-6E-6X-1 (18) |
| KC522498 | 6X |  | 6B-6E-6X-1 (18) |
| CR931643 | 7F | CR931643 (3028.3759)/CR931640 (2999..3730) | 7F-7A-1 (8) |
| JQ743531 | 7F |  | 7F-7A-1 (8) |
| JQ743532 | 7F |  | 7F-7A-1 (8) |
| JQ743533 | 7F |  | 7F-7A-1 (8) |
| JQ743534 | 7F |  | 7F-7A-1 (8) |
| JQ743535 | 7F |  | 7F-7A-1 (8) |
| JQ743536 | 7F |  | 7F-7A-1 (8) |
| JN660086 | 7F | JN660086 (1..732)/JN660115 (1..732)/JQ009436 (1..732) | 7F-21-36-1 (4) |
| JN660131 | 7F | JN660086 (1..732)/JN660115 (1..732)/JQ009436 (1..732) | 7F-21-36-1 (4) |
| CR931640 | 7A | CR931643 (3028.3759)/CR931640 (2999..3730) | 7F-7A-1 (8) |
| CR931641 | 7B | CR931641 (2666..3397)/CR931712 (3330..4061) | 7B-40-1 (1) |
| CR931642 | 7C | CR931642 (3330..4061) | 7C-1 (3) |
| JN642316 | 7C |  | 7C-1 (3) |
| JN642317 | 7C |  | 7C-1 (3) |
| CR931644 | 8 | CR931644 (3015..3746) | 8-1 (7) |
| AF316641 | 8 |  | 8-1 (7) |
| JN660132 | 8 |  | 8-1 (7) |
| JN660133 | 8 |  | 8-1 (7) |
| JN680121 | 8 |  | 8-1 (7) |
| JN680139 | 8 |  | 8-1 (7) |
| JN680144 | 8 |  | 8-1 (7) |
| AJ239004 | 8 | AJ239004 (3636..4367) | 8-2 (1) |
| CR931645 | 9A | CR931648 (3300..4031)/CR931645 (2983..3714) | 9V-9A-1 (2) |
| CR931646 | 9L | CR931646 (3302..4033) | 9L-1 (1) |
| CR931647 | 9N | CR931647 (3302..4033) | 9N-1 (3) |
| JN660134 | 9N |  | 9N-1 |
| JN660135 | 9N |  | 9N-1 |
| JN660146 | 9N | JN660146 (1..732) | 9N-2 (1) |
| AF402095 | 9V | AF402095 (2017..2748) | 9V-1 (14) |
| JN660136 | 9V |  | 9V-1 (14) |
| JN660141 | 9V |  | 9V-1 (14) |
| JN680107 | 9V |  | 9V-1 (14) |
| JN680109 | 9V |  | 9V-1 (14) |
| JN680111 | 9V |  | 9V-1 (14) |
| JN680112 | 9V |  | 9V-1 (14) |
| JN680119 | 9V |  | 9V-1 (14) |
| JN680127 | 9V |  | 9V-1 (14) |
| JN680141 | 9V |  | 9V-1 (14) |
| JQ743537 | 9V |  | 9V-1 (14) |
| JQ743538 | 9V |  | 9V-1 (14) |
| JQ743539 | 9V |  | 9V-1 (14) |
| JQ743540 | 9V |  | 9V-1 (14) |
| CR931648 | 9V | CR931648 (3300..4031)/CR931645 (2983..3714) | 9V-9A-1 (2) |
| CR931652 | 10F | CR931652 (2678..3409)/CR931651 (2592..3323) | 10F-10C-1 (3) |
| JN660088 | 10F |  | 10F-10C-1 (3) |
| CR931649 | 10A | CR931649 (2948..3679) | 10A-1 (5) |
| JN642318 | 10A |  | 10A-1 (5) |
| JN642319 | 10A |  | 10A-1 (5) |
| JN660087 | 10A |  | 10A-1 (5) |
| JN680133 | 10A |  | 10A-1 (5) |
| KC688318 | 10A | KC688318 (47..778) | 10A-2 (1) |
| CR931650 | 10B | CR931650 (2651..3382) | 10B-1 (1) |
| CR931651 | 10C | CR931652 (2678..3409)/CR931651 (2592..3323) | 10F-10C-1 (3) |
| CR931657 | 11F | CR931657 (3304..4035) | 11F-1 (1) |
| CR931653 | 11A | CR931653 (4003..4734)/CR931674 (3007..3738)/CR931656 (3329..4060) | 11A-11D-18F-1 (6) |
| CP002121 | 11A |  | 11A-11D-18F-1 (6) |
| JN642320 | 11A |  | 11A-11D-18F-1 (6) |
| JN660089 | 11A |  | 11A-11D-18F-1 (6) |
| JX102570 | 11A | JX102570 (4521..5252)/JX102571 (4525..5256) | 11A-11D-1 (3) |
| CR931654 | 11B | CR931654 (3033..3746)/CR931654 (3033..3746) | 11B-11C-1 (2) |
| CR931655 | 11C | CR931654 (3033..3746)/CR931654 (3033..3746) | 11B-11C-1 (2) |
| CR931656 | 11D | CR931653 (4003..4734)/CR931674 (3007..3738)/CR931656 (3329..4060) | 11A-11D-18F-1 (6) |
| JX102571 | 11D | JX102570 (4521..5252)/JX102571 (4525..5256) | 11A-11D-1 (3) |
| JX102572 | 11 |  | 11A-11D-1 (3) |
| CR931660 | 12F | CR931660 (2651..3382)/CR931717 (2678..3409) | 12F-44-1 (2) |
| JN660090 | 12F | CR931659(2650.3381)/JN660090 (1..732) | 12F-12B-1 (3) |
| CR931658 | 12A | CR931658 (4721..5452) | 12A-1 (1) |
| CR931659 | 12B | CR931659(2650..3381)/JN660090 (1..732) | 12F-12B-1 (3) |
| JN660091 | 12B | CR931659(2650..3381)/JN660090 (1..732) | 12F-12B-1 (3) |
| JN680126 | 12B | JN680126 (1..732) | 12B-1 (1) |
| CR931661 | 13 | CR931661 (2983..3714)/CR931679 (2983..3714) | 13-20A-20B-1 (5) |
| JQ653093 | 13 | CR931661 (2983..3714)/CR931679 (2983..3714) | 13-20A-20B-1 (5) |
| CR931662 | 14 | CR931662 (2647..3378) | 14-1 (3) |
| JN660142 | 14 |  | 14-1 (3) |
| JN660143 | 14 |  | 14-1 (3) |
| CP001033 | 14 | CP001033 (340463..341194) | 14-2 (7) |
| CP000919 | 14 |  | 14-2 (7) |
| FQ312029 | 14 |  | 14-2 (7) |
| JN660147 | 14 |  | 14-2 (7) |
| JN680130 | 14 |  | 14-2 (7) |
| JQ743542 | 14 |  | 14-2 (7) |
| JQ743541 | 14 |  | 14-2 (7) |
| X85787 | 14 | X85787 (2866..3597) | 14-3 (1) |
| JN680125 | 14 | JN680125 (1..732) | 14-4 (1) |
| JN660144 | 14 | JN660144 (1..732) | 14-5 (1) |
| CR931666 | 15F | CR931666 (3000..3731) | 15F-1 (1) |
| CR931663 | 15A | CR931663(2649..3380)/JN660108 | 15A-33B-1 (6) |
| JN660092 | 15A |  | 15A-33B-1 (6) |
| JN680113 | 15A |  | 15A-33B-1 (6) |
| JN680114 | 15A |  | 15A-33B-1 (6) |
| JN680134 | 15A |  | 15A-33B-1 (6) |
| CR931664 | 15B | CR931664 (2651..3382) | 15B-1 (2) |
| JN660093 | 15B |  | 15B-1 (2) |
| KC688319 | 15B/15C | KC688319 (55..786) | 15B/15C-1 (1) |
| CR931665 | 15C | CR931665 (2651..3382) | 15C-1 (1) |
| CR931668 | 16F | CR931668 (3005..3736) | 16F-1 (2) |
| JN660094 | 16F |  | 16F-1 (2) |
| CR931667 | 16A | CR931667 (2644..3375) | 16A-1 (1) |
| JN642321 | 17F | JN642321 (1..732) | 17F-1 (1) |
| CR931670 | 17F | CR931670 (2986..3717)/CR931700 (2652..3383) | 17F-33C-1 (5) |
| JN660137 | 17F |  | 17F-33C-1 (5) |
| JN660140 | 17F |  | 17F-33C-1 (5) |
| JN660138 | 17F |  | 17F-33C-1 (5) |
| CR931669 | 17A | CR931669 (2142..3873)/CR931703 (2902..3333) | 17A-34-1 (4) |
| JN660095 | 17A | CR931669 (2142..3873)/CR931703 (2902..3333) | 17A-34-1 (4) |
| CR931674 | 18F | CR931653 (4003..4734)/CR931674 (3007.3738)/CR931656 (3329.4060) | 11A-11D-18F-1 (6) |
| CR931671 | 18A | CR931671 (2744..3475) | 18A-1 (1) |
| CR931672 | 18B | CR931672 (2650..3381)/CR931673 (2650..3381) | 18B-18C-1 (9) |
| JN660096 | 18B |  | 18B-18C-1 (9) |
| AF316642 | 18C | AF316642 (2549.3280) | 18C-1 (1) |
| CR931673 | 18C | CR931672 (2650..3381)/CR931673 (2650..3381) | 18B-18C-1 (9) |
| JQ743543 | 18C |  | 18B-18C-1 (9) |
| JQ743544 | 18C |  | 18B-18C-1 (9) |
| JQ743545 | 18C |  | 18B-18C-1 (9) |
| JQ743546 | 18C |  | 18B-18C-1 (9) |
| JQ743547 | 18C |  | 18B-18C-1 (9) |
| JQ743548 | 18C |  | 18B-18C-1 (9) |
| CR931678 | 19F | CR931678 (4922..5653) | 19F-1 (4) |
| AF030367 | 19F |  | 19F-1 (4) |
| JF911522 | 19F |  | 19F-1 (4) |
| JF911523 | 19F |  | 19F-1 (4) |
| JF911524 | 19F | JF911524 (1448..2179) | 19F-2 (8) |
| JF911529 | 19F |  | 19F-2 (8) |
| JF911530 | 19F |  | 19F-2 (8) |
| JN642323 | 19F |  | 19F-2 (8) |
| JN642324 | 19F |  | 19F-2 (8) |
| JN642325 | 19F |  | 19F-2 (8) |
| JN664260 | 19F |  | 19F-2 (8) |
| JN680129 | 19F |  | 19F-2 (8) |
| JF911525 | 19F | JF911525 (1448..2179) | 19F-3 (8) |
| AF030368 | 19F |  | 19F-3 (8) |
| AF030369 | 19F |  | 19F-3 (8) |
| AF030370 | 19F |  | 19F-3 (8) |
| AF030372 | 19F |  | 19F-3 (8) |
| JF911526 | 19F |  | 19F-3 (8) |
| JF911527 | 19F |  | 19F-3 (8) |
| JF911531 | 19F |  | 19F-3 (8) |
| U09239 | 19F | U09239 (1616..347) | 19F-4 (1) |
| CP001015 | 19F | CP001015 (305222..305953) | 19F-5 (1) |
| JF911528 | 19F | JF911528 (1448..2179) | 19F-6 (1) |
| JN664259 | 19F | JN664259 (1..732) | 19F-7 (1) |
| JQ743553 | 19F | JQ743553 (1..732) | 19F-8 (1) |
| AF030371 | 19F | AF030371 (3407..4138)/HG799504 (1448..2179) | 19F-19A-1 (6) |
| CP000921 | 19F |  | 19F-19A-1 (6) |
| CP003357 | 19F |  | 19F-19A-1 (6) |
| CP006844 | 19F |  | 19F-19A-1 (6) |
| JQ743552 | 19F |  | 19F-19A-1 (6) |
| HG799504 | 19A | AF030371 (3407..4138)/HG799504 (1448..2179) | 19F-19A-1 (6) |
| CR931675 | 19A | CR931675 (3299..4030) | 19A-1 (2) |
| AF094575 | 19A |  | 19A-1 (2) |
| JF911512 | 19A | JF911512 (1448..2179) | 19A-2 (17) |
| CP001993 | 19A |  | 19A-2 (17) |
| HG799488 | 19A |  | 19A-2 (17) |
| HG799505 | 19A |  | 19A-2 (17) |
| JF911511 | 19A |  | 19A-2 (17) |
| JF911514 | 19A |  | 19A-2 (17) |
| JF911516 | 19A |  | 19A-2 (17) |
| JF911517 | 19A |  | 19A-2 (17) |
| JF911519 | 19A |  | 19A-2 (17) |
| JF911520 | 19A |  | 19A-2 (17) |
| JN664258 | 19A |  | 19A-2 (17) |
| JN680108 | 19A |  | 19A-2 (17) |
| JN680120 | 19A |  | 19A-2 (17) |
| JN680135 | 19A |  | 19A-2 (17) |
| JQ743549 | 19A |  | 19A-2 (17) |
| JQ743550 | 19A |  | 19A-2 (17) |
| JQ743551 | 19A |  | 19A-2 (17) |
| JF911513 | 19A | JF911513 (1448..2179) | 19A-3 (2) |
| JF911518 | 19A |  | 19A-3 (2) |
| JF911521 | 19A | JF911521 (1448..2179) | 19A-4 (2) |
| CP000936 | 19A | CP000936 (406348..407079) | 19A-5 (1) |
| JN664257 | 19A | JN664257 (1..732) | 19A-6 (1) |
| JN642322 | 19A | JN642322 (1..732) | 19A-7 (1) |
| CR931676 | 19B | CR931676 (2648..3379) | 19B-1 (1) |
| CR931677 | 19C | CR931677 (3330..4061) | 19C-1 (1) |
| CR931679 | 20 | CR931661 (2983..3714)/CR931679 (2983..3714) | 13-20A-20B-1 (5) |
| JN660097 | 20 |  | 13-20A-20B-1 (5) |
| JQ653094 | 20 |  | 13-20A-20B-1 (5) |
| CR931680 | 21 | CR931680 (3330..4061) | 21-1 (3) |
| JN642326 | 21 |  | 21-1 (3) |
| JN680140 | 21 |  | 21-1 (3) |
| JN680117 | 21B | JN680117 (1..732) | 21-2 (2) |
| JN680131 | 21B |  | 21-2 (2) |
| JQ009436 | 21 | JN660086/JN660115/JQ009436 | 7F-21-36-1 (4) |
| CR931682 | 22F | CR931682 (2983..3714)/CR931681 (2983..3714) | 22F-22A-1 (4) |
| JN660099 | 22F |  | 22F-22A-1 (4) |
| CR931681 | 22A | CR931682 (2983..3714)/CR931681 (2983..3714) | 22F-22A-1 (4) |
| JN660098 | 22A |  | 22F-22A-1 (4) |
| CR931685 | 23F | CR931685 (3329..4060) | 23F-1 (15) |
| AF030373 | 23F |  | 23F-1 (15) |
| AF030374 | 23F |  | 23F-1 (15) |
| AF057294 | 23F |  | 23F-1 (15) |
| FM211187 | 23F |  | 23F-1 (15) |
| JN664261 | 23F |  | 23F-1 (15) |
| JN680115 | 23F |  | 23F-1 (15) |
| JN680116 | 23F |  | 23F-1 (15) |
| JN680124 | 23F |  | 23F-1 (15) |
| JN680132 | 23F |  | 23F-1 (15) |
| JQ743554 | 23F |  | 23F-1 (15) |
| JQ743555 | 23F |  | 23F-1 (15) |
| JQ743556 | 23F |  | 23F-1 (15) |
| JQ743557 | 23F |  | 23F-1 (15) |
| JQ743558 | 23F |  | 23F-1 (15) |
| CR931683 | 23A | CR931683 (2651..3382) | 23A-1 (1) |
| CR931684 | 23B | CR931684 (4034..4785) | 23B-1 (3) |
| JN642327 | 23B |  | 23B-1 (3) |
| JN660100 | 23B |  | 23B-1 (3) |
| CR931688 | 24F | CR931688 (3300..4031) | 24F-1 |
| JN660102 | 24F | CR931702 (2652..3383)/CR931698 (2652..3383)/CR931704 (2677..3408)/JN660101/JN660102 (1..732) | 24F-24B-33F-33A-35A-1 (7) |
| CR931686 | 24A | CR931686 (3380..4111) | 24A-1 (1) |
| CR931687 | 24B | CR931687 (3031..3762) | 24B-1 (1) |
| JN660101 | 24B | CR931702 (2652..3383)/CR931698 (2652..3383)/CR931704 (2677..3408)/JN660101/JN660102 (1..732) | 24F-24B-33F-33A-35A-1 (7) |
| CR931690 | 25F | CR931690 (9559.10353)/CR931689 (9654..10262)/CR931710 (11116..11910) | 25F-25A-38-1 (3) |
| CR931689 | 25A | CR931690 (9559.10353)/CR931689 (9654..10262)/CR931710 (11116..11910) | 25F-25A-38-1 (3) |
| CR931691 | 27 | CR931691 (2552..3283) | 27-1 (1) |
| JN660139 | 27 | JN660139 (1..732) | 27-2 (1) |
| CR931692 | 28A | CR931693 (2675..3406)/CR931692 (2983..3714) | 28F-28A-1 (4) |
| JN660103 | 28A |  | 28F-28A-1 (4) |
| CR931693 | 28F | CR931693 (2675..3406)/CR931692 (2983..3714) | 28F-28A-1 (4) |
| JN660104 | 28F |  | 28F-28A-1 (4) |
| CR931694 | 29 | CR931694 (2650..3381) | 29-1 (2) |
| JN660105 | 29 |  | 29-1 (2) |
| CR931695 | 31 | CR931695 (3035..3766)/JN660106 (1..732) | 31-33A-1 (3) |
| JN660106 | 31 |  | 31-33A-1 (3) |
| CR931697 | 32F | CR931697 (5925..6656)/CR931696 (5824..6555) | 32F-32A-1 (2) |
| CR931696 | 32A | CR931697 (5925..6656)/CR931696 (5824..6555) | 32F-32A-1 (2) |
| CR931702 | 33F | CR931702 (2652..3383)/CR931698 (2652..3383)/CR931704 (2677..3408)/JN660101/JN660102 (1..732) | 24F-24B-33F-33A-35A-1 (7) |
| AJ006986 | 33F | CR931702 (2652..3383)/CR931698 (2652..3383)/CR931704 (2677..3408)/JN660101/JN660102 (1..732) | 24F-24B-33F-33A-35A-1 (7) |
| JN660111 | 33F | JN660111 (1..732) | 33F-1 |
| JN660107 | 33A | CR931695(3035..3766)/JN660106 (1..732) | 31-33A-1 (3) |
| CR931698 | 33A | CR931702 (2652..3383)/CR931698 (2652..3383)/CR931704 (2677..3408)/JN660101/JN660102 (1..732) | 24F-24B-33F-33A-35A-1 (7) |
| CR931699 | 33B | CR931699 (3005.3736) | 33B-1 (1) |
| JN660108 | 33B | CR931663(2649..3380)/JN660108 (1..732) | 15A-33B-1 (6) |
| CR931700 | 33C | CR931670 (2986..3717)/CR931700 (2652..3383) | 17F-33C-1 (5) |
| JN660109 | 33C | JN660109 (1..732)/JN660110 (1..732) | 33C-33D-1 (2) |
| HE651321 | 33C/33B | HE651321 (3513..4244) | 33C-1 |
| CR931701 | 33D | CR931701 (3005.3736) | 33D-1 (1) |
| JN660110 | 33D | JN660109 (1..732)/JN660110 (1..732) | 33C-33D-1 (2) |
| CR931703 | 34 | CR931669 (2142..3873)/CR931703 (2902..3633) | 17A-34-1 (4) |
| JN660112 | 34 | CR931669 (2142..3873)/CR931703 (2902..3633) | 17A-34-1 (4) |
| CR931707 | 35F | CR931707 (3304..4035)/CR931721 (3015..3746) | 35F-47F-1 (2) |
| CR931704 | 35A | CR931702 (2652..3383)/CR931698 (2652..3383)/CR931704 (2677..3408)/JN660101/JN660102 (1..732) | 24F-24B-33F-33A-35A-1 (7) |
| JN660113 | 35A |  | 24F-24B-33F-33A-35A-1 (7) |
| JN642328 | 35A | JN642328 (1..732) | 35A-1 (1) |
| CR931705 | 35B | CR931705 (2683.3414)/CR931706 (3015..3746) | 35B-35C-1 (3) |
| JN660114 | 35B |  | 35B-35C-1 (3) |
| CR931706 | 35C | CR931705 (2683.3414)/CR931706 (3015..3746) | 35B-35C-1 (3) |
| CR931708 | 36 | CR931708 (3282..4013) | 36-1 (1) |
| JN660115 | 36 | JN660086 (1..732)/JN660115 (1..732)/JQ009436 (1..732) | 7F-21-36-1 (4) |
| AJ131984 | 37 | AJ131984 (3346..4078) | 37-2 (1) |
| CR931709 | 37 | CR931709 (3034..3765) | 37-1 (1) |
| CR931710 | 38 | CR931690 (9559.10353)/CR931689 (9654..10262)/CR931710 (11116..11910) | 25F-25A-38-1 (3) |
| CR931711 | 39 | CR931711 (2679..3286) | 39-1 (1) |
| CR931712 | 40 | CR931641 (2726..3397)/CR931712 (3330..4061) | 7B-40-1 (1) |
| CR931714 | 41F | CR931714 (3345..4076) | 41F-1 (1) |
| CR931713 | 41A | CR931633 (3112.720)/CR931713 (3031..3742) | 2-41A-1 |
| CR931715 | 42 | CR931715 (2884..3615) | 42-1 (1) |
| CR931716 | 43 | CR931716 (2495..3102) | 43-1 (1) |
| CR931717 | 44 | CR931660 (2651..3382)/CR931717 (2678..3409) | 12F-44-1 (2) |
| CR931718 | 45 | CR931718 (3037.3768) | 45-1 (1) |
| CR931719 | 46 | CR931719 (2683..3414) | 46-1 (1) |
| CR931721 | 47F | CR931707 (3304..4035)/CR931721 (3015..3746) | 35F-47F-1 (2) |
| CR931720 | 47A | CR931720 (3012.3743) | 47A-1 (1) |
| CR931722 | 48 | CR931722 (2849..3580) | 48-1 (1) |

**Notes.**

*Accession number for corresponding serotype(s) reference *cpsB* sequence; the positions are the start and ending points of *cpsB* sequence in relevant GenBank sequences. Different serotypes that share the same sequetype were separate by ”/”.
