# Supplementary material for: Using a practical molecular capsular serotype prediction strategy to investigate Streptococcus pneumoniae serotype distribution and antimicrobial resistance in Chinese local hospitalized children
Source: BMC Pediatr. 2016 Apr 26;16:53. doi: 10.1186/s12887-016-0589-7 (PMC4847217; doi:10.1186/s12887-016-0589-7)
Supplement: Additional file 4: Table S3. — The cpsB sequetyping results of our 193 local isolates. (DOCX 40 kb) [file 12887_2016_589_MOESM4_ESM.docx]

**Additional file 4: Table S3. The *cpsB* sequetyping results of our 193 local isolates.**

| **Isolate ID number** | **mPCR/serotypes 6A-6D specific PCRs results** | ***cpsB* sequetype or mismatch with most closely related GenBank sequence** | **Novel *cpsB* sequetype**/**GenBank accession numbers**  **for novel *cpsB* sequence** |
| --- | --- | --- | --- |
| 230 | 3 | 1bp-3-2 | 3-sz-1(3-5)/KT164782 |
| 282 | 3 | 1bp-3-2 | 3-sz-1 (3-5)/KT164782 |
| 42 | 6/6A | 2 bp-6C-6D-1 | 6A-sz-1 (6A-5)/KT164779 |
| 73 | 6/6A | 2 bp-6C-6D-1 | 6A-sz-1 (6A-5)/KT164779 |
| 97 | 6/6A | 2 bp-6C-6D-1 | 6A-sz-1 (6A-5)/KT164779 |
| 99 | 6/6A | 2 bp-6C-6D-1 | 6A-sz-1 (6A-5)/KT164779 |
| 100 | 6/6A | 2 bp-6C-6D-1 | 6A-sz-1 (6A-5)/KT164779 |
| 220 | 6/6A | 2 bp-6C-6D-1 | 6A-sz-1 (6A-5)/KT164779 |
| 240 | 6/6A | 2 bp-6C-6D-1 | 6A-sz-1 (6A-5)/KT164779 |
| 242 | 6/6A | 2 bp-6C-6D-1 | 6A-sz-1 (6A-5)/KT164779 |
| 244 | 6/6A | 2 bp-6C-6D-1 | 6A-sz-1 (6A-5)/KT164779 |
| 267 | 6/6A | 2 bp-6C-6D-1 | 6A-sz-1 (6A-5)/KT164779 |
| 276 | 6/6A | 2 bp-6C-6D-1 | 6A-sz-1 (6A-5)/KT164779 |
| 148 | 6/6A | 2 bp-6C-6D-1 | 6A-sz-1 (6A-5)/KT164779 |
| 288 | 6/6B | 6B-1 |  |
| 219 | 6/6C | 6C-6D-1 |  |
| 307 | 6/6C | 6C-6D-1 |  |
| 21 | 6/6B | 6B-6E-6X-1 |  |
| 40 | 6/6B | 6B-6E-6X-1 |  |
| 52 | 6/6B | 6B-6E-6X-1 |  |
| 69 | 6/6B | 6B-6E-6X-1 |  |
| 75 | 6/6B | 6B-6E-6X-1 |  |
| 105 | 6/6B | 6B-6E-6X-1 |  |
| 109 | 6/6B | 6B-6E-6X-1 |  |
| 281 | 6/6B | 6B-6E-6X-1 |  |
| 286 | 6/6B | 6B-6E-6X-1 |  |
| 297 | 6/6B | 6B-6E-6X-1 |  |
| 299 | 6/6B | 6B-6E-6X-1 |  |
| 313 | 6/6B | 6B-6E-6X-1 |  |
| 238 | 9V/9A | 9V-1 |  |
| 298 | 9V/9A | 9V-1 |  |
| 308 | 9V/9A | 9V-1 |  |
| 19 | 14 | 14-1 |  |
| 300 | 14 | 14-1 |  |
| 83 | 14 | 14-1 |  |
| 91 | 14 | 14-1 |  |
| 136 | 14 | 14-1 |  |
| 137 | 14 | 14-1 |  |
| 138 | 14 | 14-1 |  |
| 229 | 14 | 14-1 |  |
| 277 | 14 | 14-1 |  |
| 295 | 14 | 14-1 |  |
| 301 | 14 | 14-1 |  |
| 306 | 14 | 14-1 |  |
| 309 | 14 | 14-1 |  |
| 310 | 14 | 14-1 |  |
| 214 | 15F/15A | 3bp-15A-33B-1 | 15F/15A-sz-1(15F-15A-1)*******/**KT164781 |
| 293 | 15F/15A | 3bp-15A-33B-1 | 15F/15A-sz-1(15F-15A-1) ***/**KT164781 |
| 72 | 15B/15C | 15B/15C-1 |  |
| 87 | 15B/15C | 15B/15C-1 |  |
| 107 | 15B/15C | 15B/15C-1 |  |
| 256 | 15B/15C | 15B/15C-1 |  |
| 259 | 15B/15C | 15B/15C-1 |  |
| 312 | 15B/15C | 15B/15C-1 |  |
| 29 | 15B/15C | 15B/15C-1 |  |
| 34 | 15B/15C | 15B/15C-1 |  |
| 113 | 15B/15C | 15B/15C-1 |  |
| 123 | 15B/15C | 15B/15C-1 |  |
| 213 | 15B/15C | 15B/15C-1 |  |
| 215 | 15B/15C | 15B/15C-1 |  |
| 216 | 15B/15C | 15B/15C-1 |  |
| 5 | 19F | 19F-19A-1 |  |
| 6 | 19F | 19F-19A-1 |  |
| 9 | 19F | 19F-19A-1 |  |
| 20 | 19F | 19F-19A-1 |  |
| 22 | 19F | 19F-19A-1 |  |
| 23 | 19F | 19F-19A-1 |  |
| 24 | 19F | 19F-19A-1 |  |
| 25 | 19F | 19F-19A-1 |  |
| 26 | 19F | 19F-19A-1 |  |
| 31 | 19F | 19F-19A-1 |  |
| 43 | 19F | 19F-19A-1 |  |
| 54 | 19F | 19F-19A-1 |  |
| 56 | 19F | 19F-19A-1 |  |
| 58 | 19F | 19F-19A-1 |  |
| 61 | 19F | 19F-19A-1 |  |
| 66 | 19F | 19F-19A-1 |  |
| 67 | 19F | 19F-19A-1 |  |
| 68 | 19F | 19F-19A-1 |  |
| 76 | 19F | 19F-19A-1 |  |
| 81 | 19F | 19F-19A-1 |  |
| 86 | 19F | 19F-19A-1 |  |
| 88 | 19F | 19F-19A-1 |  |
| 94 | 19F | 19F-19A-1 |  |
| 124 | 19F | 19F-19A-1 |  |
| 289 | 19F | 19F-19A-1 |  |
| 292 | 19F | 19F-19A-1 |  |
| 106 | 19F | 19F-19A-1 |  |
| 112 | 19F | 19F-19A-1 |  |
| 115 | 19F | 19F-19A-1 |  |
| 117 | 19F | 19F-19A-1 |  |
| 120 | 19F | 19F-19A-1 |  |
| 122 | 19F | 19F-19A-1 |  |
| 125 | 19F | 19F-19A-1 |  |
| 126 | 19F | 19F-19A-1 |  |
| 133 | 19F | 19F-19A-1 |  |
| 211 | 19F | 19F-19A-1 |  |
| 222 | 19F | 19F-19A-1 |  |
| 223 | 19F | 19F-19A-1 |  |
| 224 | 19F | 19F-19A-1 |  |
| 225 | 19F | 19F-19A-1 |  |
| 227 | 19F | 19F-19A-1 |  |
| 232 | 19F | 19F-19A-1 |  |
| 235 | 19F | 19F-19A-1 |  |
| 239 | 19F | 19F-19A-1 |  |
| 241 | 19F | 19F-19A-1 |  |
| 243 | 19F | 19F-19A-1 |  |
| 247 | 19F | 19F-19A-1 |  |
| 248 | 19F | 19F-19A-1 |  |
| 249 | 19F | 19F-19A-1 |  |
| 251 | 19F | 19F-19A-1 |  |
| 252 | 19F | 19F-19A-1 |  |
| 254 | 19F | 19F-19A-1 |  |
| 257 | 19F | 19F-19A-1 |  |
| 258 | 19F | 19F-19A-1 |  |
| 260 | 19F | 19F-19A-1 |  |
| 262 | 19F | 19F-19A-1 |  |
| 265 | 19F | 19F-19A-1 |  |
| 269 | 19F | 19F-19A-1 |  |
| 270 | 19F | 19F-19A-1 |  |
| 271 | 19F | 19F-19A-1 |  |
| 273 | 19F | 19F-19A-1 |  |
| 284 | 19F | 19F-19A-1 |  |
| 287 | 19F | 19F-19A-1 |  |
| 291 | 19F | 19F-19A-1 |  |
| 302 | 19F | 19F-19A-1 |  |
| 304 | 19F | 19F-19A-1 |  |
| 305 | 19F | 19F-19A-1 |  |
| 17 | 19A | 19A-2 |  |
| 38 | 19A | 19A-2 |  |
| 49 | 19A | 19A-2 |  |
| 64 | 19A | 19A-2 |  |
| 65 | 19A | 19A-2 |  |
| 74 | 19A | 19A-2 |  |
| 90 | 19A | 19A-2 |  |
| 93 | 19A | 19A-2 |  |
| 101 | 19A | 19A-2 |  |
| 129 | 19A | 19A-2 |  |
| 131 | 19A | 19A-2 |  |
| 217 | 19A | 19A-2 |  |
| 218 | 19A | 19A-2 |  |
| 245 | 19A | 19A-2 |  |
| 253 | 19A | 19A-2 |  |
| 272 | 19A | 19A-2 |  |
| 275 | 19A | 19A-2 |  |
| 278 | 19A | 19A-2 |  |
| 280 | 19A | 19A-2 |  |
| 283 | 19A | 19A-2 |  |
| 285 | 19A | 19A-2 |  |
| 290 | 19A | 19A-2 |  |
| 311 | 19A | 19A-2 |  |
| 234 | 20 | 13-20A-20B-1 |  |
| 255 | 20 | 13-20A-20B-1 |  |
| 10 | 23F | 23F-1 |  |
| 45 | 23F | 23F-1 |  |
| 77 | 23F | 23F-1 |  |
| 77 | 23F | 23F-1 |  |
| 80 | 23F | 23F-1 |  |
| 103 | 23F | 23F-1 |  |
| 104 | 23F | 23F-1 |  |
| 108 | 23F | 23F-1 |  |
| 118 | 23F | 23F-1 |  |
| 128 | 23F | 23F-1 |  |
| 212 | 23F | 23F-1 |  |
| 226 | 23F | 23F-1 |  |
| 228 | 23F | 23F-1 |  |
| 237 | 23F | 23F-1 |  |
| 246 | 23F | 23F-1 |  |
| 274 | 23F | 23F-1 |  |
| 294 | 23F | 23F-1 |  |
| 141 | 23F | 23F-1 |  |
| 142 | 23F | 23F-1 |  |
| 149 | 23F | 23F-1 |  |
| 16 | 23F | 11bp-11F-1 | 23F-sz-1 (23F-2)/KT164778 |
| 35 | 23F | 11bp-11F-1 | 23F-sz-1 (23F-2)/KT164778 |
| 37 | 23F | 11bp-11F-1 | 23F-sz-1 (23F-2)/KT164778 |
| 135 | 23F | 11bp-11F-1 | 23F-sz-1 (23F-2)/KT164778 |
| 221 | 23F | 11bp-11F-1 | 23F-sz-1 (23F-2)/KT164778 |
| 261 | 23F | 11bp-11F-1 | 23F-sz-1 (23F-2)/KT164778 |
| 263 | 23F | 11bp-11F-1 | 23F-sz-1 (23F-2)/KT164778 |
| 264 | 23F | 11bp-11F-1 | 23F-sz-1 (23F-2)/KT164778 |
| 279 | 23F | 11bp-11F-1 | 23F-sz-1 (23F-2)/KT164778 |
| 296 | 23F | 2bp-6A-6B-6F-1 | 23F-sz-2 (23F-2)/KT164783 |
| 32 | 23F | 6A-6B-6F-1 | 23F-6A-6B-6F-sz-1/KT164777 |
| 233 | 23F | 6A-6B-6F-1 | 23F-6A-6B-6F-sz-1/KT164777 |
| 144 | 23F | 13-20A-20B-1 | 23F-13-20A-20B-1/KT164780 |
| 266 | 23A | 23A-1 |  |
| 314 | 23A | 23A-1 |  |
| 236 | 34 | 17A-34-1 |  |
| 231 | unknown | 10B-1 |  |
| 78 | unknown | 28F-28A-1 |  |
| 250 | unknown | unknown |  |
| 268 | unknown | unknown |  |
