# Supplementary material for: Using a practical molecular capsular serotype prediction strategy to investigate Streptococcus pneumoniae serotype distribution and antimicrobial resistance in Chinese local hospitalized children
Source: BMC Pediatr. 2016 Apr 26;16:53. doi: 10.1186/s12887-016-0589-7 (PMC4847217; doi:10.1186/s12887-016-0589-7)
Supplement: Additional file 5: Table S4. — Serotype distribution by clinical presentation among our 193 S. pneumonie isolates from children. (DOC 44 kb) [file 12887_2016_589_MOESM5_ESM.doc]

**Additional file 5: Table S4. Serotype distribution by clinical presentation among our 193 *S. pneumonie* isolates from children**

| **Serotype** | **No. (%) of isolates** | | | | | |
| --- | --- | --- | --- | --- | --- | --- |
|  | **Pneumonia**  **(n=172)** | | **Bacteremia**  **(n=17)** | **Meningitis**  **(n=2)** | **Other**  **(n=2)** | **Total**  **(n=193)** |
|  | **Non-severe pneumonia**  **(n=134)** | **Severe pneumonia**  **(n=38)** |  |  |  |  |
| 19F | 47 (35.0) | 14 (36.8) | 6 (30) | 0 (0.0) | 1 (50) | 67 (34.7) |
| 23F | 22 (16.4) | 8 (22.9) | 3 (15) | 0 (0.0) | 0 (0.0) | 33 (17.1) |
| 19A | 15 (11.2) | 6 (15.7) | 3 (15) | 0 (0.0) | 0 (0.0) | 23 (11.9) |
| 14 | 7 (5.2) | 2 (5.7) | 4 (20) | 1 (50) | 0 (0.0) | 14 (7.3) |
| 15B/15C | 11 (9.0) | 2 (5.7) | 0 (0.0) | 0 (0.0) | 0 (0.0) | 13 (6.7) |
| 6B | 12 (9.0) | 0 (0.0) | 1 (5.0) | 0 (0.0) | 0 (0.0) | 13 (6.7) |
| 6A | 8 (6.0) | 3 (8.6) | 0 (0.0) | 1 (50) | 0 (0.0) | 12 (6.2) |
| Other | 12 (9.0) | 3 (7.9) | 0 (0.0) | 0 (0.0) | 1 (50) | 18 (9.3) |

Note. No significant differences were detected between serotype and clinical presentation.
