# Supplementary material for: Using a practical molecular capsular serotype prediction strategy to investigate Streptococcus pneumoniae serotype distribution and antimicrobial resistance in Chinese local hospitalized children
Source: BMC Pediatr. 2016 Apr 26;16:53. doi: 10.1186/s12887-016-0589-7 (PMC4847217; doi:10.1186/s12887-016-0589-7)
Supplement: Additional file 6: Table S5. — Percentages of resistant to antibiotics for serotypes with 10 or more isolates. (DOC 36 kb) [file 12887_2016_589_MOESM6_ESM.doc]

**Additional file 6: Table S5. Percentages of resistant to antibiotics for serotypes with 10 or more isolates**

| **Serotype** | **No. of non-meningitis isolates** | **% of** **non-meningitis isolates resistant to** | | |
| --- | --- | --- | --- | --- |
|  |  | **Penicillin** | **Ceftriaxone** | **Erythromycin** |
| 19F | 67 | 1.5 | 14.9 | 97.0 |
| 23F | 33 | 3.0 | 0 | 96.9 |
| 19A | 23 | 0 | 0 | 95.6 |
| 14 | 13 | 0 | 0 | 100 |
| 15B/15C | 13 | 0 | 0 | 100 |
| 6B | 13 | 0 | 0 | 100 |
| 6A | 11 | 0 | 0 | 90.9 |
| Others | 18 | 0 | 0 | 100 |
| Total | 191 | 1.0 | 5.2 | 97.4 |
